# Supramolecular Assembly of Graphene–Polyamine–PdS–CdS Photocatalysts for Synergistically Enhanced and Highly Effective Hydrogen Evolution from Water under Visible Light

**DOI:** 10.1021/acs.inorgchem.5c02898

**Published:** 2025-10-07

**Authors:** María L. Godino-Salido, Francesco Bartoli, Alba M. Valbuena-Rus, Giuseppe Vicidomini, María D. Gutiérrez-Valero, Victor K. Abdelkader-Fernández, Matteo Savastano, Antonio Bianchi, Rafael López-Garzón

**Affiliations:** † Department of Inorganic and Organic Chemistry, Faculty of Experimental Sciences, Jaén University, 23071 Jaén, Spain; ‡ 201843 Institute of Chemistry of Organometallic Compounds-National Research Council of Italy, Via Madonna Del Piano 10, 50019 Sesto Fiorentino, Florence, Italy; § Department of Chemistry “Ugo Schiff”, 201779 University of Florence, 50019 Sesto Fiorentino, Florence, Italy; ∥ Department of Inorganic Chemistry, Faculty of Sciences, Granada University, 18071 Granada, Spain; ⊥ Department for the Promotion of Human Science and Quality of Life, 441756 University San Raffaele Roma, 00166 Rome, Italy; ∇ National Interuniversity Consortium of Materials Science and Technology (INSTM), Research Unit of Florence, Via G. Giusti 9, 50121 Florence, Italy

## Abstract

New composite materials for water reduction based on the CdS photocatalyst were prepared by sequential deposition of semiconductor PdS and CdS on a platform obtained from graphene nanoplatelets (GNPs) noncovalently functionalized by the adsorption of the [9]­aneN_3_ pyrimidine derivatives HL1 and H_2_L2. The GNP-HL1-PdS-CdS composite (≈ 4.5, 0.5, 95 wt % composition) showed excellent photocatalytic activity in water reduction with an almost constant average H_2_ production of 4.05 mmol·g^–1^·h^–1^ during 28 h, overcoming the best-performing analogous composites reported so far. The systematic study of the structural and optical properties of both the composites and the precursors reveals that besides PdS and CdS, GNP-HL1 behaves as a 2D semiconductor. The excellent performance of the GNP-HL1-PdS-CdS composite in water reduction is explained by the suitable alignment of VB and CB of CdS, acting as a photocatalyst, to those of both PdS and GNP-HL1, acting as cocatalysts. The small size of GNP sheets, together with the chemical nature of HL1 functions, facilitates the light activation of the sites of the photocatalysts, resulting in effective water reduction at the surface of both CdS and GNP-HL1 components.

## Graphical abstract

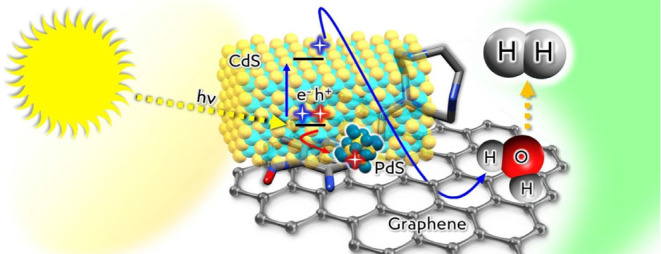

## 1 Introduction

As the dramatic effects of greenhouse gases on climate change become more and more evident, reducing the global use of fossil fuels continues to be a crucial challenge to the society. Thus, the development of renewable and sustainable energy sources is being boosted as a priority research goal to face this social and environmental concern.^1,2^ The Sun is an endless source of energy for the Earth's surface (in the time scale of human beings) that largely exceeds the worldwide energy utilization.^3–6^ Even though solar energy is difficult to handle directly, it can be conveniently converted and stored into other forms, including chemical ones.^7–9^ Among these, it is found that the dihydrogen molecule (H_2_) is a convenient fuel in different technologies,^10,11^ whose combustion produces water as the only reaction product. H_2_ can be obtained by total or partial splitting of the water molecule by photocatalytic redox processes in which semiconductors acting as catalysts become activated by solar light as the energy source. Light activation of the photocatalysts occurs by promoting electrons from their valence band (VB) to the conduction band (CB), resulting in the formation of hole–electron pairs. In order to achieve the total redox decomposition of the water molecule, the energy of the CB band of the semiconductor should be below 0.00 eV and that of the VB should be higher than 1.23 eV (in the NHE scale).^12^ Thus, a minimum semiconductor bandgap of 1.23 eV is necessary to accomplish the total splitting of water into its elemental components, H_2_ and O_2_. In practice, the required bandgap is larger due to kinetic barriers affecting the process (mainly oxygen oxidation) and the associated overpotential.

A wide range of photocatalytic metal-based semiconductors, capable of producing H_2_ and/or O_2_ from H_2_O under light irradiation, including sulfides, nitrides, single metal oxides, and double metal oxides (perovskites), have been successfully used in water reduction reaction.^3,13–15^ Among them, CdS has attracted great interest due to both the bandgap value, ca. 2.4 eV, and the suitable position of the CB.^16–18^

An important factor limiting the catalytic performance of semiconductor catalysts is the quick recombination of the hole–electron pairs. As a strategy to prevent recombination, the thickness of CdS crystals has been reduced so that charge carriers can reach the surface faster.^19,20^ Moreover, suitable control of the thickness of CdS crystals also allows tailoring its bandgap, as it increases with the decrease of crystal thickness.^21–23^ Nevertheless, in the face of fast electron–hole recombination, the efficiency of these strategies is quite limited. A more successful strategy to improve CdS performance in water splitting is its coupling with a different 2D semiconductor.^24,25^ The semiconductor of choice must have a CB bottom energy suitable to accept electrons from CdS, thus stabilizing the charge separation in the material. As a matter of fact, coupling of partially oxidized graphene oxide GO and other graphene compounds to different metal-based semiconductors increases their photocatalytic performance.^17,26^

In a previous study,^27^ we have reported that the photocatalytic performance of CdS in water reduction reaction under solar radiation is significantly increased when CdS crystals are deposited on a graphene (G) platform decorated with a triamine ligand (labeled as Tren, see Scheme 1). G is a 2D conducting material that becomes a 2D semiconductor when its extended sp^2^ surface network is partially blocked by the adsorption of pyrimidine derivatives (Scheme 1). Moreover, control of the amount of the adsorbed pyrimidine compound allows the fine-tuning of G bandgap.^27,28^ The work function of pristine graphene, lying in its VB, almost levels that of hydrogen;^3,29^ thus, the splitting of its VB and CB, resulting in enhancement of the energy at the bottom of its CB, should favor the reduction ability of the excited electrons transferred from the surface of CdS toward H_2_ generation. Therefore, when combined with CdS, both the high surface area of suitably functionalized G and the high mobility of the electrons transferred to it from CdS are key factors significantly contributing to the improvement of the photocatalytic performance.

**Scheme 1:**
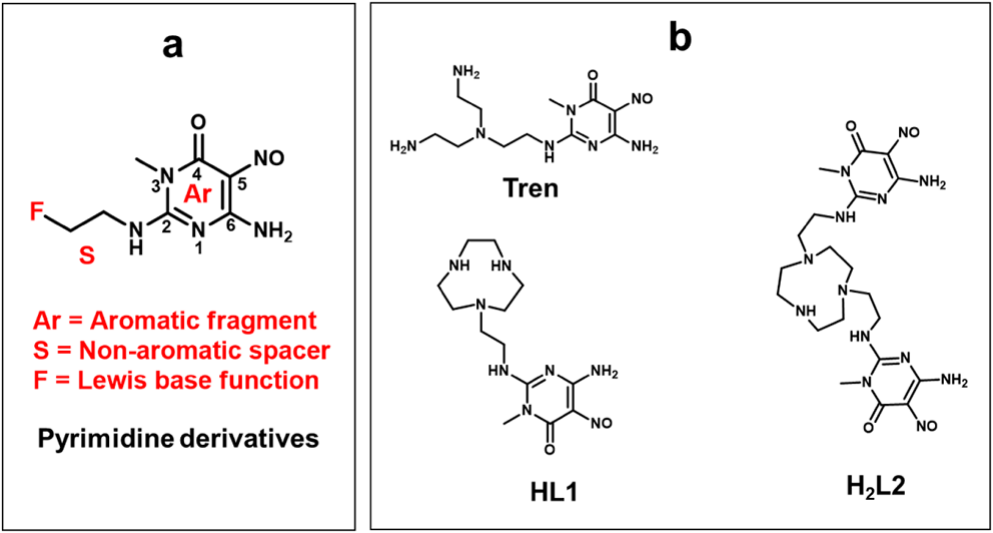
Schematic Representation of Pyrimidine Derivatives Used for Noncovalent Functionalization of Graphene Surfaces: a) General Formula; b) HL1 and H_2_L2 Derivatives (Used in This Study) and Tren (Used in a Previous Work)^27^

It must be mentioned that Cd­(II) is a concerning metal cation due to its inherent toxicity and bioaccumulation possibility. While CdS is also a pollutant agent that may seriously affect the human health upon entering the body via breathing or ingestion,^30^ it is also known that CdS insolubility increases its in vivo tolerability. In any case, developing strategies to prevent the release of CdS under using of CdS-based photocatalysts is relevant.^31^ In the case of the above-mentioned G-Tren-CdS photocatalyst,^27^ a triamine function attached to the C(2) position of a pyrimidine moiety of Tren contributes to stabilize CdS nanoparticles deposited on G-Tren surface, thereby preventing, to a certain extent, the release of environmentally toxic CdS when using the photocatalyst. The use of a suitable sacrificial agent in the photocatalytic reaction (a Na_2_S/Na_2_SO_3_ mixture) also contributes to prevent the release of Cd via corrosion of the photocatalyst.

In the above-mentioned G-Tren CdS-based photocatalyst,^27^ the semiconductor CdS (acting as a photocatalyst) was mixed with PdS as a dopant (cocatalyst). In the resulting PdS-CdS mixture (with ca. PdS 0.5 wt %), PdS acts as an efficient stabilizer of the hole–electron pairs created in CdS under light irradiation, thus improving the performance of pristine CdS in the reaction.^32^ Moreover, the very high absorption coefficient of PdS allows it to work efficiently even if the amount loaded in the mixture is very small, thus compensating for its relatively high cost.^33^

Furthermore, the above study showed that the photocatalytic performance in water reduction of PdS-CdS was greatly improved when this mixture was deposited on the graphene platform G-Tren (obtained by adsorption of the Tren derivative, Scheme 1, on the G surface).

The enhanced photocatalytic activity shown by the resulting G-Tren-PdS-CdS material was attributed to the fact that the electrons excited from the VB to the CB of CdS under light radiation are easily transferred to the CB of G-Tren.^27,34–36^ Thus, if the energy at the bottom of the CB of the semiconductor G-Tren (which can be controlled as it is a function of the coverage of Tren on G) is tailored to a suitable value, the composite results are very effective in the water splitting process.

It was shown for the G-Tren-PdS-CdS photocatalyst the existence of important attractive interactions between the sheets of the G-Tren platforms, due both to the contribution of intersheet hydrogen bonds between the branched aliphatic triamine functions and to van der Waals interactions between G sheets of relatively large size.^27^ Extensive stacking of the sheets should limit light penetration to the inner photocatalytic centers,^17^ thus worsening the photocatalytic performance.

This last aspect suggested that a suitable redesigning of the G platform, aimed at minimizing the stacking effect while preserving its ability to hybridize with both CdS and PdS nanoparticles, should produce a significant improvement of the photocatalytic performance. With this aim, a new photocatalyst, GNP-HL1-PdS-CdS, was prepared by sequential deposition of PdS and CdS nanoparticles on the new GNP-HL1 graphene platform. This substrate has been obtained from commercial graphene nanoplatelets (GNPs), whose surface has been functionalized by adsorption of the HL1 molecule shown in Scheme 1. GNP was chosen as a graphene-type matrix because its chemical nature (extended sp^2^ moiety) and high surface area, analogous to G,^27,37^ make it a similarly good cocatalyst candidate to pair with CdS. The much lower size of GNP sheets (mean size <1.5 μm) vs G (mean size <10 μm) should contribute to reducing the observed and undesired trend to sheet stacking. On the other side, instead of Tren, the HL1 ligand was adsorbed on GNP to obtain the 2D-semiconductor platform. Both Tren and HL1 consist of the same conjugate pyrimidine moiety (same effect on G bandgap) although the arm at its C (2) position (Scheme 1) is much less prone to the formation of hydrogen bonds in HL1 with respect to the branched aliphatic triamine of Tren.

In this work, the preparation and systematic characterization of GNP-HL1-PdS-CdS and its precursors have been carried out, together with the study of their photocatalytic activity in water reduction. The studies show the excellent performance of GNP-HL1-PdS-CdS in the mentioned reaction. Faced with almost a doubling of performance with respect to G-Tren-PdS-CdS (2.30 mmol·g^–1^·h^–1^,^27^ vs 4.05 mmol·g^–1^·h^–1^ for the new catalyst), herein we provide further insights about the main factors subtending to it (reduced stacking, bandgap control, ability of HL1 ligand to stabilize PdS nanoparticles). It is noteworthy that the above results greatly improve those shown by similar photocatalysts as GO-CdS, g-C_3_N_4_–CdS, and g-C_3_N_4_–Pd-CdS composites in the same reaction.^38–40^ Therefore, this points out that, in designing G-based CdS photocatalysts, strategies that minimize stacking of graphene sheets and control of the CB energy of the graphene moiety to a suitable position relative to the CB of CdS, are key points to optimize the performance of photocatalysts in water reduction. Suitable alignment of the CB of the graphene relative to the CB of the CdS is key to allow electron transfer from the latter to the surface of graphene where the high electron mobility considerably enhances water reduction.

To gain even further insights into the role played by the choice of HL1 ligand for the photocatalytic performance of GNP-HL1-PdS-CdS, a similar material was prepared and studied: GNP-H_2_L2-PdS-CdS, where H_2_L2 consists of an analogous HL1 molecule (Scheme 1). GNP-H_2_L2-PdS-CdS still shows good photocatalytic behavior in water reduction, although significantly lower (c.a. 29%) than GNP-HL1-PdS-CdS. The difference has been related to the more extensive blocking of the sp^2^ surface of the functionalized GNP in the case of H_2_L2 molecule, indicating the delocalized G-type surface as a key active center for water reduction.

## 2 Experimental Section

### 2.1 Materials

The commercial GNP used in this work, supplied by Nanografi (Ankara, Turkey), consists of stacks of graphene sheets (<10 units, average diameter <1.5 μm). Characterization of GNP from XPS data was done by our group in a previous work.^37^ Briefly, GNP sheets contain C (92.2 wt %, mainly corresponding to graphene Csp^2^) and O (7.8 wt %), which is found as epoxy, carbonyl, carboxyl, and hydroxyl functions.^37^

Before use, GNP was suspended in water under stirring for 24 h to remove the labile oxygen groups and then separated by filtration and air-dried.

All of the solvents and the other chemicals were of analytical grade and used without any further purification.

### 2.2 Ligand Synthesis

The synthesis of precursors 2-(1,4,7-triazonan-1-yl)­ethanamine and 2,2’-(1,4,7-triazonane-1,4-diyl)­diethanamine was performed as previously described.^41^

Their functionalization with 6-amino-3,4-dihydro-3-methyl-2-methoxy-5-nitroso-4-oxopyrimidine to obtain HL1 and H_2_L2 ligands was performed according to the described procedures.^42^ The ligands were isolated as solid products. Yields of the functionalization step were 92.1% for HL1 and 83.8% for H_2_L2.

HL1: ^1^H NMR (D_2_O, 400 MHz, pH 4.0) δ 3.75 (t, 2 H), 3.64 (s, 4 H), 3.42 (s, 3 H), 3.35 (t, 4 H), 3.14 (t, 4 H), 3.03 (t, 2 H). Elemental analysis: calcd. For HL1·MeOH·2.5 H_2_O (C_14_H_33_N_8_O_5.5_) C, 41.88; H, 8.29; N, 27.91; exp 41.74; H 6.53; N 28.21.

H_2_L2: ^1^H NMR (D_2_O, 400 MHz, pH 1.0) = δ 3.30 (m, 4 H); 3.36 (s, 6 H); 3.40 (m, 4H); 3.49 (m, 8H); 3.95 (m, 4H). Elemental analysis: calcd. For H_2_L2 (C_20_H_33_N_13_O_4_) C, 46.23; H, 6.40; N, 25.05; exp 45.97; H 6.34; N 24.94.

### 2.3 Ligand Protonation and Metal-Binding Properties

Ligand protonation and metal complexation constants toward Cu­(II) and Cd­(II) were determined via potentiometric methods according to previously described procedures.^43^ Cu­(II) has been used as a test cation considering that (i) Pd­(II) complexation could not be studied due to its typical kinetic sluggishness, (ii) Cu­(II) typically forms the most stable complexes among divalent first-row transition metals, and (iii) Pd­(II) complexes are known to be much more stable than Cu­(II) ones. Typically, titrations were performed in 0.1 M NMe_4_Cl aqueous solution at 298.1 ± 0.1 K using an automated apparatus. Emf data acquisition was performed with the computer program PASAT.^44,45^ The combined electrode (Metrohm 6.0262.100) was calibrated as a hydrogen-ion concentration probe by titration of known amounts of HCl with CO_2_-free NaOH solutions and determining the equivalent point by Gran’s method,^46^ which furnished the standard potential, E°, and the ionic product of water (pKw = 13.83(1) in 0.1 M NMe_4_Cl at 298.1 K). The concentration of ligand was about 1 × 10^–3^ M in all experiments, and the studied pH range was ≈2.0–11.5. Data treatment was performed with the HYPERQUAD software.^47^

Protonation and Pd­(II) binding events have also been monitored via UV–vis absorption spectra recorded at 298.1 K by using a Jasco V-670 spectrophotometer. Ligands spectra were recorded in the pH range 0.5–12 at a concentration of 5.0 x 10^–5^ M (HL1) or 2.0 x 10^–5^ M (H_2_L2). UV–vis spectra of the Pd­(II) complex were recorded in the pH range 2–10. A stock solution containing ligand and metal ion (concentrations given above) was prepared at pH 3, to avoid formation of metal hydroxides/oxides, and allowed to equilibrate. The UV–vis spectrum of this solution was checked daily until an invariance was reached. This stock solution was used to prepare solutions at different pH values (2–10), which were allowed to equilibrate for 8 days. The recorded pH of these solutions was measured after the equilibrium was reached.

### 2.4 Preparation of the Photocatalysts

Both the solid tested as photocatalysts and those used as starting materials in this work have been prepared under sustainable experimental conditions (room temperature and water as solvent), according to a previously reported methodology.^27^ The final photocatalysts GNP-HL1-PdS-CdS and GNP-H_2_L2-PdS-CdS have been prepared by the sequential deposition of PdS and CdS NPs on a matrix consisting of GNP supramolecularly functionalized by adsorption of a pyrimidine derivative (GNP-HL1 or GNP-H_2_L2), as illustrated in Scheme 2. Details of the experimental conditions used for the preparation of all materials are included in the Supporting Information.

**Scheme 2:**
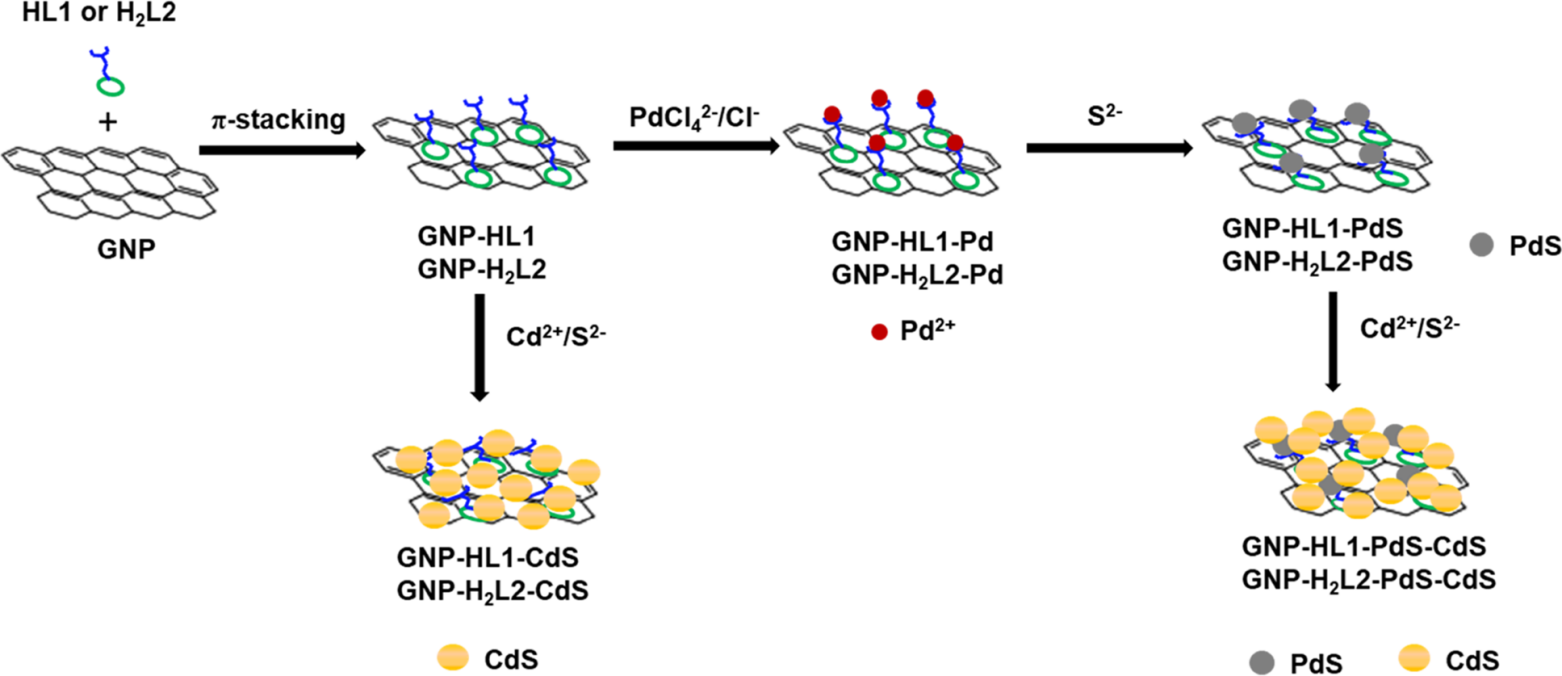
Schematic Representation of the Preparation Procedure of Studied Photocatalysts

### 2.5 Characterization of the Materials

X-ray diffraction (XRD) of the materials was performed at room temperature on a Panalytical Empyrean diffractometer using Cu Kα radiation (λ = 1.5406 Å) over a 2θ angular range of 10–90°, at a scan rate of 5° min^–1^.

UV–vis absorption spectra of the samples were obtained in water dispersions whenever possible, in the 200–1000 nm range, by using 1 cm quartz cells on a PerkinElmer Lambda 25 spectrophotometer. Once the corresponding spectra were obtained, the suspensions corresponding to samples including HL1 in their composition were filtered off, and the spectra of the corresponding filtrates were obtained to ensure that no significant desorption of HL1 had taken place. In the case of the less dispersible GNP-HL1-PdS-CdS sample, the UV–visible diffuse reflectance spectra (DRS) were obtained on a Shimadzu UV-2600 spectrophotometer, over the 300–800 nm range, using BaSO_4_ as a reference. Bandgap energies of both direct and indirect electron transitions were calculated by analyzing the Tauc plots resulting from the Kubelka–Munk transformation of the UV–visible spectra.^48^

The samples were analyzed by photoluminescence spectroscopy (PL) in an Edinburgh Instruments FLS920 spectrofluorometer, using a 450 W Xe lamp (Xe900) as the light source. The spectra, obtained by irradiating the solid samples at 425 nm, exhibit low emission values, probably due to the tiny particle size of the sample components (PdS, CdS, and GNP-HL1 nanoparticles), which could determine a high density of surface defects that quench emission from the excitons.^49^ Attempts to obtain PL spectra from water dispersions were also made but were discarded due to their drastically reduced emission signal.

The textural characterization of GNP and GNP-HL1 samples based on N_2_ and CO_2_ adsorption measurements was carried out using volumetric adsorption equipment ASAP 2020 (Analyzer of Surface Area and Porosity from Micromeritics). N_2_ isotherms were registered at 77 K and CO_2_ isotherms at 273 K. In both cases, the samples were previously outgassed under vacuum conditions at 90 °C for 12 h. The specific surface areas and the micropore volumes were determined applying the Brunauer–Emmett–Teller (BET) and the Dubinin–Radushkevich equations, respectively.

X-ray photoelectron spectra (XPS) were obtained in a Kratos Axis Ultra DLD spectrometer. Monochromatic Al/MgKα radiation in constant analyzer energy mode with pass energies of 160 and 20 eV (for the survey and high-resolution spectra, respectively) was used. The C 1s transition at 284.6 eV was used as a reference to obtain the heteroatom binding energies. The accuracy of the binding energy (BE) values was ± 0.2 eV.

The morphology of the materials was studied by transmission electron microscopy (TEM), using FEI Talos F200X equipment (Thermo Fisher Scientific, Waltham, MA, USA) that combined outstanding high-resolution TEM and STEM with an HAADF imaging with industry-leading energy-dispersive X-ray spectroscopy (EDX) signal detection and chemical characterization with compositional mapping.

### 2.6 Photocatalytic Experiments

The photocatalytic hydrogen production experiments were performed at 298.1 K and atmospheric pressure in a 140 mL double-walled flask, sealed with a silicone rubber septum. A solar simulator (PICO G2 V Optics, Inc.), providing a standard AM1.5G light spectrum, was used as a visible light source to trigger the photocatalytic reaction under solar simulated conditions. In a typical experiment, 5 mg of the catalyst was dispersed in an aqueous solution (5 mL) of 0.35 M Na_2_S and 0.25 M Na_2_SO_3_ as sacrificial agent. A continuous magnetic stirrer was applied at the bottom of the reactor in order to keep the photocatalyst in a suspension. Prior to irradiation, the system was bubbled with argon for 20 min to remove the dissolved oxygen. During the irradiation process, 0.02 mL of the generated gas was collected through the septum for analysis at predesigned time intervals. The hydrogen content in the generated gas was analyzed by gas chromatography (GC-Agilent Technologies 7820A, TCD, Ar carrier, 5 Å molecular sieve column). The apparent quantum efficiency (AQE) of the H_2_ evolution was measured with a 420 nm high-power LED as a light source. For this experiment, the photoreactor (containing 30 mg of photocatalyst in 5 mL of 0.35 M Na_2_S and 0.25 M Na_2_SO_3_ aqueous solution at 298.1 K and atmospheric pressure) was placed 1 cm away from the LED source. The moles of incident photons in unit time (1.8·10^–7^ einstein·s^–1^) were measured by chemical actinometry, using potassium ferrioxalate as actinometer.^50,51^ The AQE (%) was calculated according to the following equation:

$$\mathrm{AQE}\;(\%)=\frac{\mathrm{number of reacted electrons in unit time}}{\mathrm{number of incident photons in unit time}}\times 100$$

$$=\frac{2\times \mathrm{number of evolved}{\;H}_{2}\;\mathrm{molecules in unit time}}{\mathrm{number of incident photons in unit time}}\times 100$$

## 3 Results and Discussion

### 3.1 Acid–Base Properties of the HL1 and H_2_L2 Ligands

Table 1 lists the determined stability constants for the protonation of HL1 and H_2_L2, as well as for the formation of their Cu­(II) and Cd­(II) complexes.

**Table 1:** Determined Protonation and Metal Binding Constants for the HL1 and H_2_L2 Ligands (*I* = 0.1 M NMe_4_Cl, T = 298 K)^a^

	log*K*		
Equilibrium	-	Cu(II)	Cd(II)
L1^–^ + H^+^ = HL1	11.2(1)		
HL1 + H^+^ = H_2_L1^+^	10.94(1)		
H_2_L1^+^ + H^+^ = H_3_L1^2+^	5.73(6)		
H_3_L1^2+^ + H^+^ = H_4_L1^3+^	2.13(8)		
H_4_L1^3+^ + H^+^ = H_5_L1^4+^	1.8(1)		
L1^–^ + M^2+^ = [ML1]^+^		21.62(2)	15.65(1)
HL1 + M^2+^ = [MHL1]^2+^		15.93(6)	12.58(5)
H_2_L1^+^ + M^2+^ = [MHL1]^3+^			6.87(2)
[L1M]^+^ + 2 OH^–^ = [ML1(OH)_2_]^−^		6.99(6)	7.77(8)
L2^2–^ + H^+^ = HL2^–^	11.84(4)		
HL2^–^ + H^+^ = H_2_L2	11.19(4)		
H_2_L2 + H^+^ = H_3_L2^+^	10.12(3)		
H_3_L2^+^ + H^+^ = H_4_L2^2+^	3.84(5)		
H_4_L2^2+^ + H^+^ = H_5_L2^3+^	2.21(5)		
H_5_L2^3+^ + H^+^ = H_6_L2^4+^	2.30(5)		
L2^2–^ + M^2+^ = [ML2]		18.0(2)	17.9(1)
HL2^–^ + M^2+^ = [MHL2]^+^		16.3(1)	15.23(7)
H_2_L2 + M^2+^ = [MH_2_L2]^2+^		11.77(5)	11.93(4)
H_3_L2^+^ + M^2+^ = [MH_3_L2]^3+^		5.49(4)	5.56(4)
H_4_L2^2+^ + M^2+^ = [MH_4_L2]^4+^		3.1(3)	-
L2^2–^ + M^2+^ + OH^–^ = [ML2(OH)]^−^		20.6(1)	22.69(8)

a Values in parentheses are the standard deviation of the last significant figure

Regarding their (Brønsted-Lowry) basicity, both ligands present 3 protonable sites of high, intermediate, and low basicity, congruent with the presence of the [9]­aneN_3_ macrocyclic moiety. As expected, HL1 (2 secondary amines and 1 tertiary amine in the macrocycle) is found to be more basic than H_2_L2 (1 secondary amine and 2 tertiary amines instead). As previously discussed for related derivatives, insertion of each pyrimidine residue implies 2 further acid–base equilibria. These are namely the deprotonation of the NH group linked to the C(2) of the pyrimidine residue to yield anionic species, in a very alkaline (pH > 11) medium, and the protonation of the NO group of the pyridine, in a very acidic (pH ≈ 2) medium. This assignment of the protonation sites is supported by the pH-dependence of the UV–vis bands of both ligands, which is only sensitive to the first and the last protonation equilibria as they concern the aforementioned sites close to the pyrimidine chromophore.

Related UV–vis spectra and species distribution diagrams are reported in the Figures S1 and S2.

### 3.2 Metal-Binding Properties of the HL1 and H_2_L2 Ligands

As mentioned, Cu­(II) was employed as the test cation. Overall, data confirm that the two ligands retain the well-known favorable binding properties of the [9]­aneN_3_ macrocycle. Data show (see distribution diagrams in Figure S3) that in a 1:1 M:L solution, there is significant free Cu­(II) cation only at pH < 4 for H_2_L2 (2 tertiary amines and 1 secondary amine in the macrocycle) and at pH < 3 for HL1 (1 tertiary amine and 2 secondary amines in the macrocycle) (cf. acid–base section).

Pd­(II) complexes are expected and found to be more stable than Cu­(II) ones. Although Pd­(II) binding constants could not be determined due to the typical kinetic inertness of the metal ion, UV–vis spectra of 1:1 M:L equilibrium solutions clearly show that the ligand is already engaged in Pd­(II) binding below pH 2 and remains so over all the explored pH range (up to ≈ 11) (Figure S4) for both ligands. This proves that HL1 and H_2_L2 are fully capable of binding Pd­(II) ions.

The same goes for Cd­(II) ions (distribution diagrams are reported in Figure S3), whose complexes are almost equally stable with H_2_L2, and slightly less stable with HL1, with respect to the analogous Cu­(II) species.

According to the above and to the key concerns of the present study, we can conclude that both HL1 and H_2_L2 are capable of effective binding toward Pd­(II) and Cd­(II) ions, as later exploited for the growth/deposition of nanosized PdS or CdS crystals. Given the inherent basicity of the ligands and the acidity of the metal centers at the surface of nanosized semiconductors, it is reasonable to expect (see below) that the ligands at the surface can act as stabilizing agents toward the deposited nanoparticles.

### 3.3 Preparation and Characterization of the Photocatalysts

In previous studies, a series of both Pd-based catalysts and CdS-based photocatalysts were prepared by supporting them on G and GNP previously functionalized with a series of C(2)-substituted derivatives of 6-amino-3,4-dihydro-3-methyl-5-nitroso-4-oxopyrimidine, analogous to the compound HL1 used in this work.^27,37,52^ It has been found that the adsorptivity of this class of pyrimidine ligands on GNP overcomes that of G.^37^ High adsorptivity on GNP of the above type of derivatives offers a considerable advantage in catalysis because (i) it leads to obtain catalysts with a greater density of the metal active sites as these are linked by the complexing functions at C(2) of the pyrimidine moiety,^27^ (ii) the higher the range of ligand adsorptivity, the wider the range in which the bandgap of GNP ligand can be tuned,^27,28^ (iii) the reduced size of GNP sheets compared to the G should minimize the blocking of the catalytic active centers. Due to these reasons, GNP was chosen as the starting material in this work. The choice of HL1 (Scheme 1) to functionalize GNP was made as it was expected that the macrocyclic triamine should have a lessened tendency to form intersheet H-bonds with respect to the branched triamine of Tren, thus diminishing the stacking of GNP-HL1 sheets, detrimental for the performance of the photocatalyst.^27^ These expectations are based upon the known leaning of macrocycles (especially if small) toward intramolecular hydrogen bonds and upon the diminished basicity of HL1 (Table 1) with respect to Tren^53^ (as a qualitative parameter for hydrogen bond propensity). GNP-HL1-PdS-CdS was obtained according to the general procedure described in Scheme 2. Both the general procedure and the characterization of the intermediates are detailed in the following sections. The H_2_L2 ligand was devised at a later stage of the study in order to address a few questions concerning mechanistic aspects of photocatalysis, as described in the dedicated section.

#### 3.3.1 Preparation and Characterization of GNP-HL1

GNP-HL1 hybrid was obtained through a suitable adsorption experiment on GNP of HL1 in aqueous solution (see Supporting Information), which reveals the maximum adsorption capacity of 0.77 mmol·g^–1^ (Figure S5a). This value is similar to the adsorptivity of analogous previously studied pyrimidine derivatives on GNP,^37^ and is notably higher than the values found when G was used as adsorbent instead.

Studies on the adsorption of a large number C(2)-substituted derivatives of 6-amino-3,4-dihydro-3-methyl-5-nitroso-4-oxopyrimidine analogous to HL1 on carbon substrates having in common graphene surfaces (activated carbons,^54–58^ carbon nanotubes,^37,42,59^ graphene^28,37,52^ and graphene nanoplatelets^37^) indicate that the adsorption of this kind of ligands takes place through strong π–π interactions of the electron-deficient pyrimidine moiety of the ligand with the basic arene centers (Cπ) of the graphene surface. Such interactions are significantly strong and are driven by van der Waals forces reinforced by an electrostatic component.^42,60^ The above π–π interactions cause significant shifts of the binding energy (BE) values of the N 1s and O 1s components in the XPS spectra of the pyrimidine moiety of the free ligands after their adsorption on the graphene support. In fact, desorption of HL1 from GNP-HL1 is very low (Figure S5a), indicating that the ligand is strongly retained on the surface of GNP.

In the XPS spectra in Figure 1, the N 1s signal of HL1 contains two components: the component of lower BE value (397.4 eV) corresponds to the three nitrogen atoms of the cyclic triamine ring, and that at higher energy (398.7 eV) corresponds to the five nitrogen atoms conjugated with the pyrimidine moiety.

**Figure 1:**
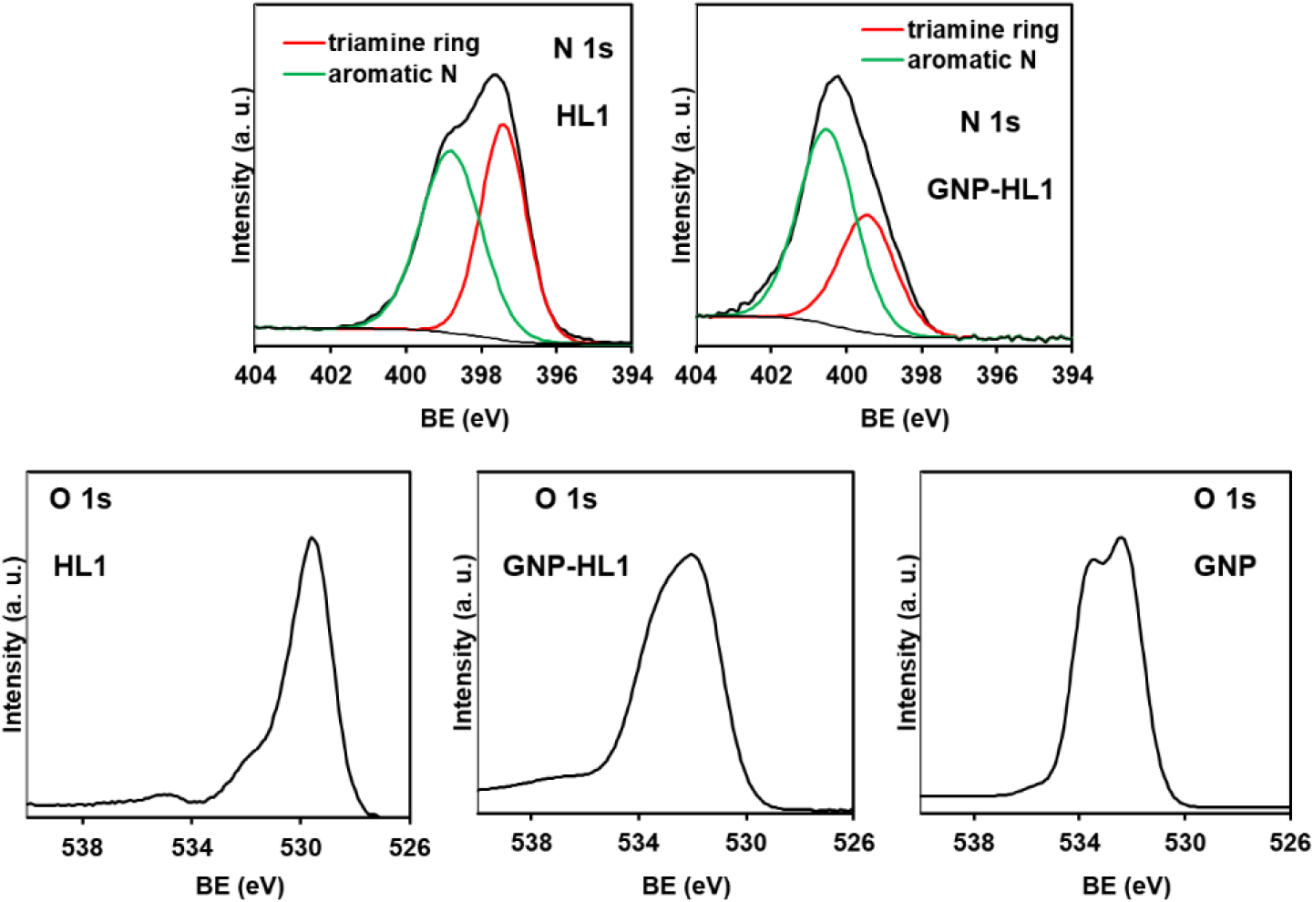
High-resolution XPS spectra in the N 1s region of HL1 and GNP-HL1, and in the O 1s region of HL1, GNP-HL1, and GNP.

Lack of nitrogen in the composition of GNP means that the N 1s spectrum of GNP-HL1 in Figure 1 is entirely due to HL1 molecules of the hybrid. The component of the aromatic nitrogen atoms is placed at 400.6 eV, and that of the triamine ring at 399.3 eV. The wide shifting to higher BE values of the aromatic nitrogen atoms of HL1 upon adsorption on GNP is similar to that observed in other analogous cases and is due to Cπ–π_pyr_ interactions of the Cπ centers and the pyrimidine moieties. The interacting moieties are compressed one onto the other causing local repulsion of the adjoined π-clouds of both giving rise to deshielding of the N and O aromatic atoms of the pyrimidine. The N 1s component of the three nonaromatic nitrogen atoms of the hybrid also shifts with respect to that of the free ligand, likely due to the fact that HL1-HL1 interactions in solid HL1 are different from HL1-GNP ones. Involvement of the pyrimidine in solid-state ligand–ligand interactions has been observed for analogous cases.^53,60^ A similar behavior to that described for the aromatic component of N 1s is followed by the O 1s signal of the conjugated C(5)­NO and C(6)O groups when HL1 is adsorbed on GNP. This signal, at ca. 529.3 eV in the spectrum of the bare HL1 (Figure 1), shifts under adsorption on GNP to higher BE values up to the range in which the O 1s signal of GNP is found (ca. 530–536 eV, see Figure 1), becoming hidden by the latter.

The XPS data discussed above point out that the adsorption of HL1 on GNP takes place mainly by the C_π_–π_pyr_ interaction of the arene centers of the surface graphene and the planar pyrimidine moiety of HL1. Thus, other possible mechanisms (i.e., those implying interactions of functional groups of HL1 and oxygen functions of GNP) could be discarded. The interaction of C_π_–π_pyr_ type should keep the metal complexing ability of the HL1 functions, so favoring stabilization of metal particles further deposited on GNP surface as observed in other similar cases (graphitized ACs,^55,58^ MWCNTs,^43,59^ and G^28^).

Textural characteristics of heterogeneous photocatalysts are also relevant, as they can affect catalytic performance. As said, ligand–ligand interlayer H-bonding and π–π stacking among G sheets reduce light penetration toward inner catalytic centers and overall efficiency of the catalyst.^17^ The Scherrer equation was applied^61,62^ to the well-defined peak at 2θ = 26.5 ° in the X-ray diffractograms of GNP and GNP-HL1 (Figure S6) which correspond to the diffraction from the slits between the packed sheets of each of the materials. Average values of the size (height) of the stacked units existing in the GNP and GNP-HL1 were thus obtained (Table S1). The calculated values, 8.63 nm for pristine GNP and 8.12 nm for GNP-HL1, indicate that the functionalization of GNP with HL1 does not induce significant changes in the stacking. Still, calculation of BET surface areas from the corresponding N_2_ isotherms (Figure S7) shows that the surface area of GNP-HL1 (201.0 m^2^·g^–1^) is largely lower than the GNP one (605.3 m^2^·g^–1^).

Moreover, the pore volume associated with micropores (obtained from the corresponding CO_2_ isotherms, Figure S8) also decreases under functionalization of GNP with HL1 from 0.129 cm^3^·g^–1^ to 0.057 cm^3^·g^–1^. The fact that functionalization of GNP does not induce enhancement of sheet stacking but reduces the BET surface area and micropore volume can be tentatively explained as a result of increasing cross-linking/lateral interactions between stacked-sheet blocks, resulting in a denser packing.

Analyzing the optical properties of GNP-HL1 is important, as such a platform is an active player in the photocatalytic water reduction by the GNP-HL1-PdS-CdS catalyst. Accordingly, the UV–visible spectra of GNP and GNP-HL1 were obtained from the corresponding aqueous suspensions (Figure 2a).

**Figure 2:**
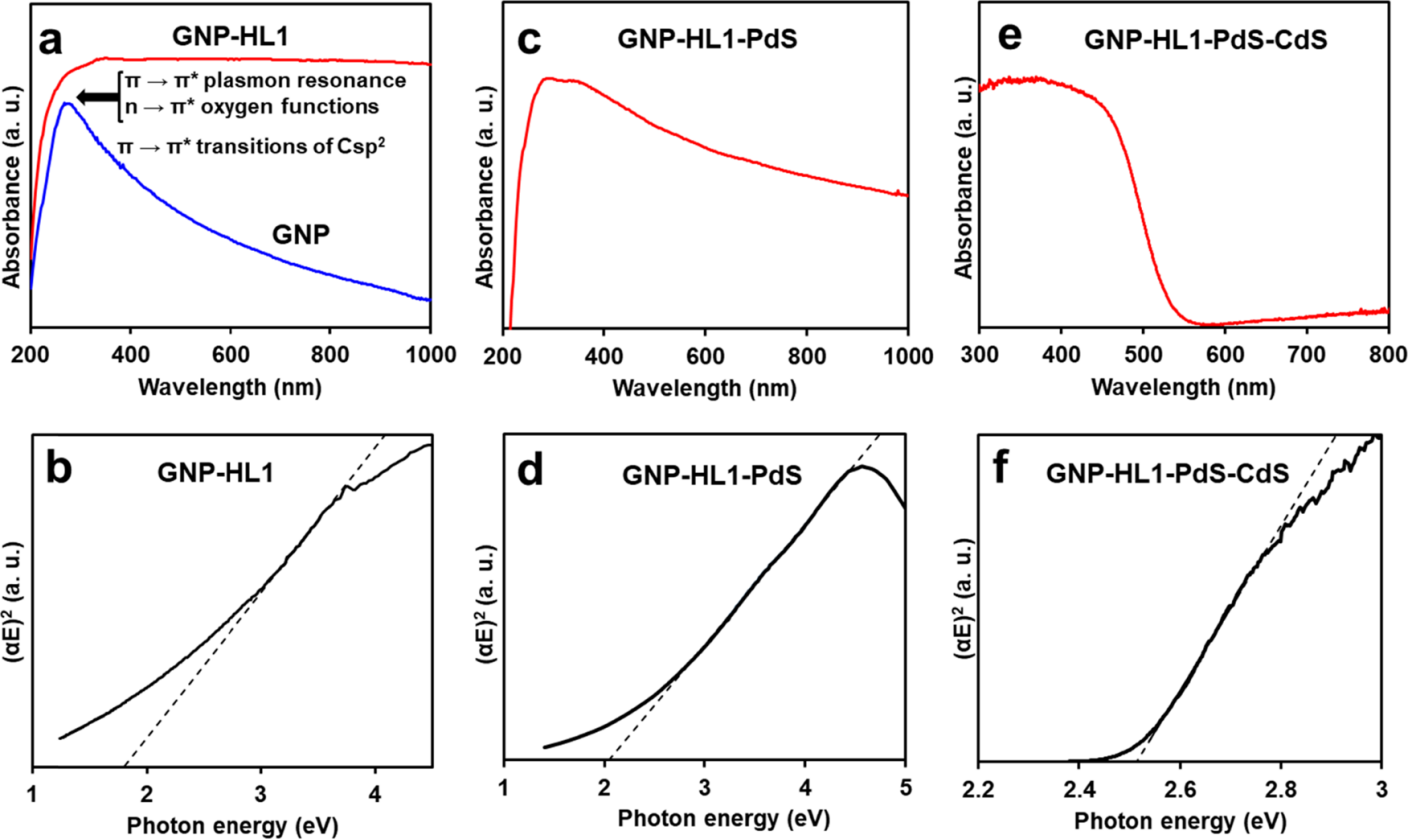
UV–vis absorption spectra of a) GNP and GNP-HL1 hybrid; c) GNP-HL1-PdS; e) GNP-HL1-PdS-CdS. Plots of (αE)^2^ (direct transitions) vs photon energy (eV) for b) GNP-HL1 hybrid; d) GNP-HL1-PdS; and f) GNP-HL1-PdS-CdS.

Spectrum of GNP in the UV range shows a wide band with maximum at ca. 280 nm, which is typical of π → π* plasmon resonance in sp^2^ domains of graphene surface. The asymmetry of the band is due to n → π* transitions of the oxygen functions which should appear at ca. 300–320 nm,^63–65^ and the absorption extended in the 400–800 nm range is due to electron π → π* transitions of the Csp^2^ domains. Spectrum of GNP-HL1 shows higher absorption both in the 300–400 nm UV range and in the whole visible range. In the case of the UV range, it is due to the high absorptivity of HL1 (Figure S1), which sums to that of GNP. Enhancement of the absorption in the visible range upon adsorption of pyrimidine derivatives induces splitting of the VB and CB of the pristine G, introducing an additional absorption due to the electronic transitions between the bands. In other words, the splitting should arise from the partial blocking of the C 2p_*z*_ electrons of GNP surface,^42,53,55,56,66^ resulting in the formation of isolated sp^2^ domains of reduced and quite uniform sizes, whose VB and CB become separated by an energy gap. It has been previously observed that the higher the amount of ligand adsorbed per graphene surface unit, the larger the gap between the VB and the CB; such effect has been explained as the higher surface coverage the lesser the size of sp^2^ domains generated upon adsorption.^27,28^

Tauc plots of Figures 2b and S9 were obtained from the corresponding UV–visible spectra. Those corresponding to indirect transitions, (αE)^1/2^ vs the photon energy (where α = absorbance and E = photon energy), do not show any defined absorption edge, neither for pristine GNP nor for GNP-HL1. On the other hand, the plots for direct transitions, (αE)^2^ vs the photon energies, do not display any absorption edge only in the case of GNP, indicating a lack of an energy gap between the VB and CB in this material. On the contrary, the (αE)^2^ vs hυ plot of GNP-HL1 (Figure 2b) shows defined absorption edges due to a direct transition at 1.8 eV. Having in mind that HL1 lacks any absorption due to direct transitions in the visible range, the one observed for GNP-HL1 hybrid is attributed to the splitting of VB and CB of GNP under the adsorption of HL1.

#### 3.3.2 Preparation and Characterization of GNP-HL1-Pd

According to Scheme 2, the GNP-HL1-PdS-CdS catalyst was prepared from GNP-HL1 by consecutive deposition of (i) minor amounts of PdS cocatalyst, obtaining the GNP-HL1-PdS hybrid, and (ii) the CdS catalyst, to obtain GNP-HL1-PdS-CdS. The final material has an approximate 4.5, 0.5, 95 wt % composition of GNP-HL1, PdS and CdS, respectively. As said, PdS was selected as the dopant semiconductor due to the suitable alignment of its VB and CB to that of CdS, which helps delaying the recombination of the photogenerated electron–hole pair.^27,28^ Moreover, the very high absorption coefficient of PdS allows high effectiveness even if the added amount to CdS photocatalyst is small (<0.5 wt %), thus compensating its relatively high cost.^33,67^ Deposition of PdS on the surface of GNP-HL1 was done by adsorption of PdCl_4_^2–^, followed by subsequent addition of the equimolar amount of Na_2_S to an aqueous suspension of the obtained hybrid, namely GNP-HL1-Pd (0.60 mmol·g^–1^ of Pd). Although PdCl_4_^2–^ shows very low adsorptivity on pristine GNP (c.a. 0.08 mmol·g^–1^), this increases significantly when GNP-HL1 is used as adsorbent (Figure S5b). This is due to the complexation ability toward Pd­(II) (see above) of the [9]­aneN_3_ function of adsorbed HL1. Similar adsorptivity enhancements were observed for analogous derivatives bearing different complexing functions.^27,37^ It is known that the [9]­aneN_3_ ligand, unable to fulfill a square planar coordination geometry, behaves as a bidentate ligand toward Pd­(II) ions (e.g., as per the VUYGEC,^68^ ERAQOE,^69^ and related crystal structures), as also done by some small N4 macrocycles that cannot adjust to such geometry (we recently reported the PEKRIK and PEKROQ crystal structures).^37^ Adsorption of Pd­(II) on GNP-HL1 does not induce any noticeable change of the BE of the N 1s component of the triamine ring (see Figures 1 and 3a). It is worth noticing that the macrocycle is expected to be diprotonated at pH 5 (Figure S1, H_3_L1^2+^ species >80% formation), so that the metal cation replaces the acidic protons. The appearance of a narrow doubly peaked signal in the XPS spectrum of GNP-HL1-Pd due to 2 p_3/2_ (198.0 eV) and 2 p_1/2_ (199.6 eV) states of equivalent Cl^–^ ions (Figure 3b) suggests these act probably also as auxiliary ligands of the complexed Pd­(II) ions (as per the above-mentioned crystal structures evidence). However, the N 1s component of the aromatic nitrogen of HL1 splits into two components in the XPS spectrum of GNP-HL1-Pd; the higher intensity one is placed at similar BE with respect to the precursor (Figure 1) and the weaker one appears at a higher BE (ca. 402.5 eV, see Figure 3a). This last component is tentatively assigned to the C(5)­NO grouping of the pyrimidine moiety lying on the GNP plane. Again, crystal structures evidence how this group can bind metal cations.^70–73^ As a matter of fact, either via proper coordination or as a proximity effect, the adsorbed pyrimidine clearly senses the presence of Pd­(II) ions. Concerning Pd oxidation state, two symmetric peaks assignable to Pd, at 338.0 and 343.2 eV in the XPS spectrum of GNP-HL1-Pd (due to 3 d_5/2_ and 3 d_3/2_ states, Figure 3c) indicate that most of the adsorbed Pd is oxidized.^74^ Neither X-ray diffractogram nor TEM images of GNP-HL1-Pd (Figures S6 and 4) provide any evidence of the existence of Pd(0) nanoparticles on the surface. The presence of two peaks at 3.5 and 5.0 eV in the VB region of the XPS spectrum of GNP-HL1-Pd (Figure 3d) should be assigned to Pd­(II) in different chemical environments. The elemental composition (at %) of GNP-HL1-Pd obtained from the survey XPS spectrum (Table S2) yields an atomic relationship N/Pd ≈ 8, therefore an HL1/Pd = 1, suggesting the whole Pd (II) is adsorbed by complexation with HL1 molecules. This points out that no Pd(0) is found in GNP-HL1-Pd which is supported by the lack of any nanoparticles in the HRTEM image (Figure 4a). In fact, the BE value reported for Pd(0) in this range (c.a. 2.0 eV)^75^ is quite lower than those of the peaks observed in the case of GNP-HL1-Pd. The presence of two peaks in the VB XPS spectrum of GNP-HL1-Pd is probably due to Pd­(II) adsorbed via different complexation patterns to HL1. In fact, at the pH value for the preparation of GNP-HL1-Pd (pH 5, see Supporting Information), both HL1 and L1 can act as different ligand species to Pd­(II) (Figures S2 and S3) which would require two different auxiliary ligands to Pd­(II) (i.e., C(5)­NO group of HL1 and/or Cl^–^). The last is consistent with the Cl/Pd atom relationship found for the GNP-HL1-Pd material, 1.53 (see Table S2), which is lower than 2.

**Figure 3:**
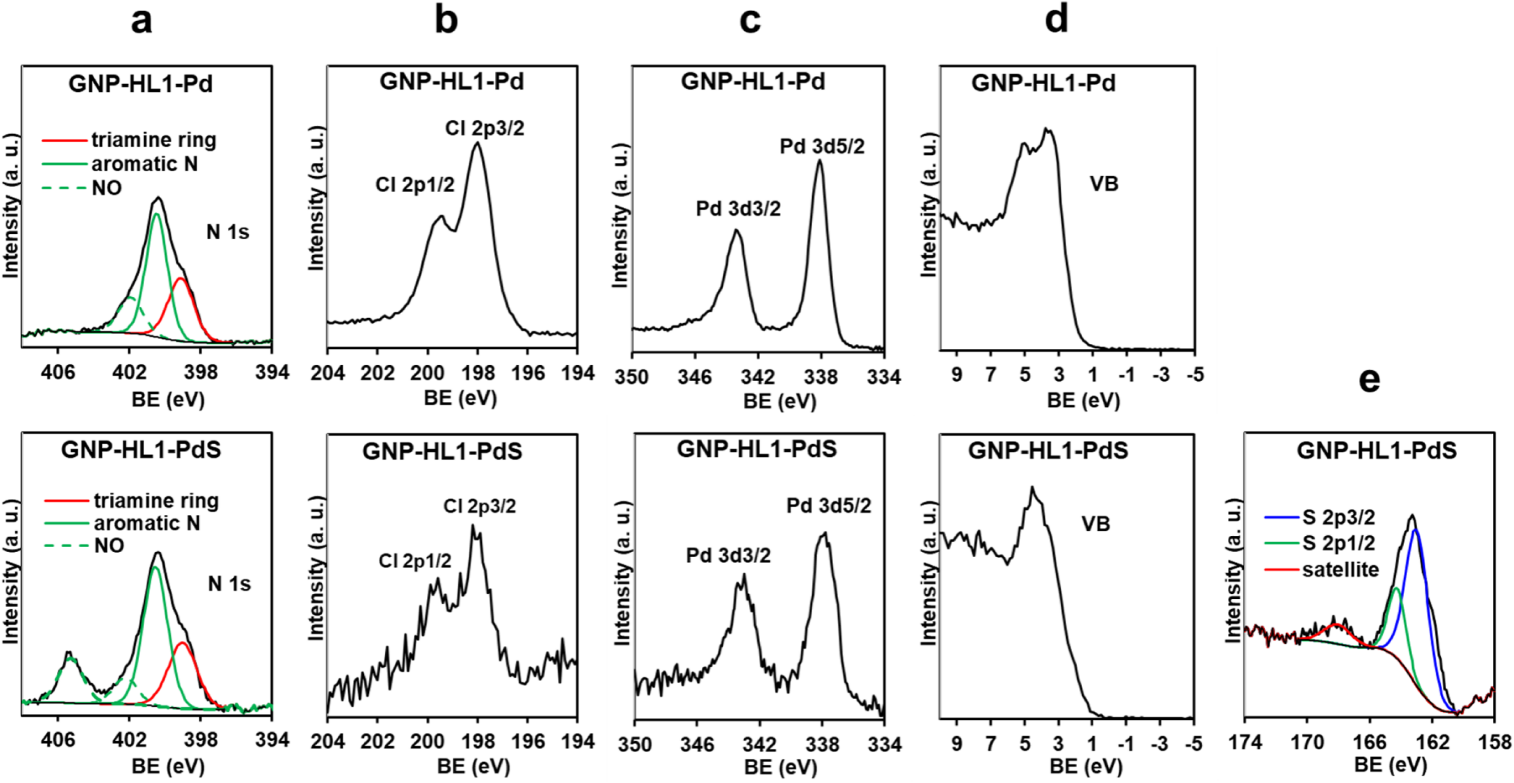
High-resolution XPS of GNP-HL1-Pd and GNP-HL1-PdS in the a) N 1s, b) Cl 2p, c) Pd 3d, d) VB, and e) S 2p regions.

**Figure 4:**
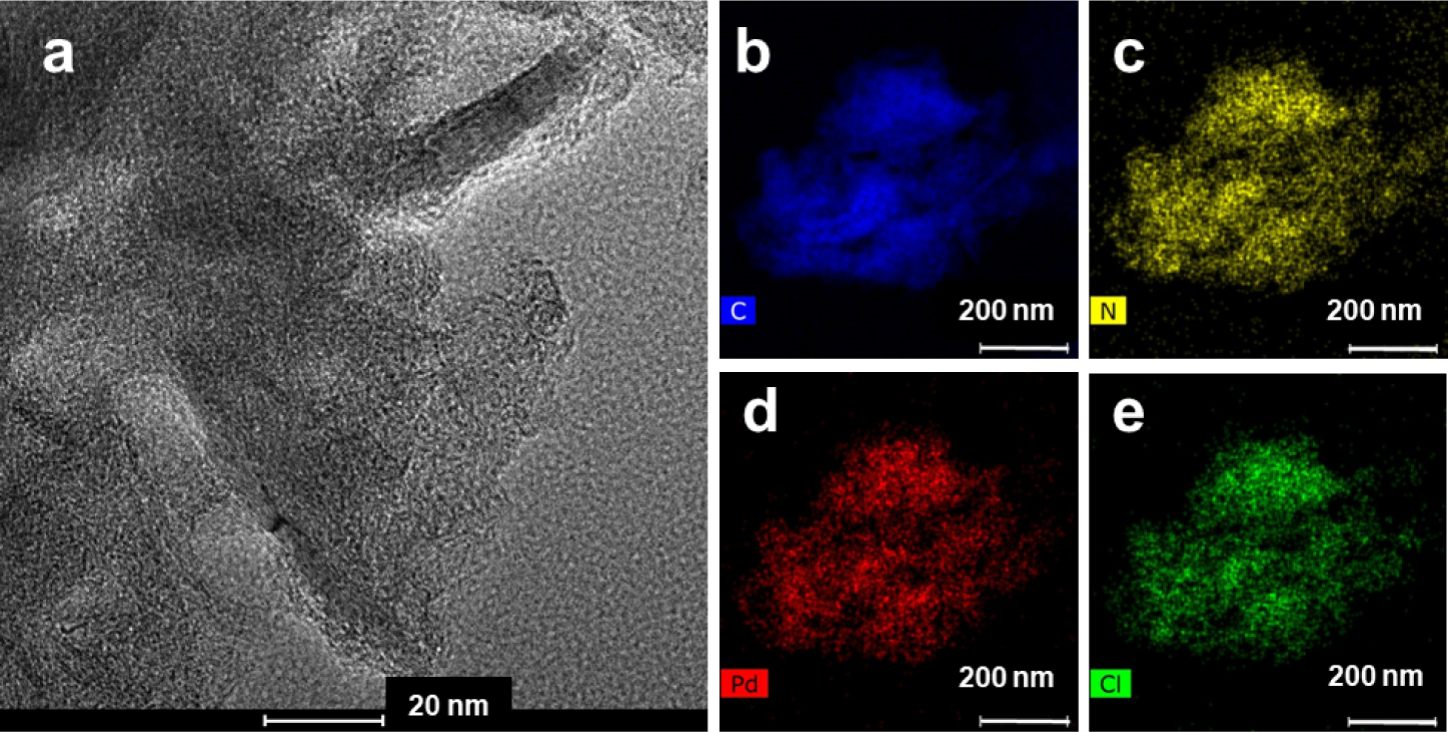
HRTEM image (a) and element distribution mapping of GNP-HL1-Pd (b: carbon, c: nitrogen, d: palladium, and e: chlorine).

Element distribution mapping of GNP-HL1-Pd (Figure 4) shows a very uniform distribution of N (namely, of HL1) and also of both Pd and Cl ions on the GNP surface, as expected assuming Pd­(II) is adsorbed via metal–ligand complexation.

#### 3.3.3 Preparation and Characterization of GNP-HL1-PdS

According to the literature,^76^ precipitation of PdS from solutions containing stable Pd­(II) complexes under addition of S^2–^ strongly favors the formation of PdS nanoparticles with a very small size, meaning both high surface area of the cocatalyst and improved light absorptivity. Slow addition of a stoichiometric amount of Na_2_S to a water suspension of GNP-HL1-Pd should result in almost quantitative precipitation of Pd­(II) as PdS, on the basis of the very high p*K*_sp_ value (p*K*_spPdS_ = 60.1).^77^ HRTEM and HAADF-STEM images of Figure 5 show uniform spreading of very few nanoparticles (less than 2 nm) on the GNP surface component. Elements mapping in Figure 5 is consistent with GNP (carbon) acting as the matrix support for dispersed Pd, S, and N components. Moreover, the distribution patterns of these three elements are similar, which is consistent with the possible role of the triamine cyclic function of the adsorbed HL1 in stabilizing the formed PdS nanoparticles (see the paragraph below).

**Figure 5:**
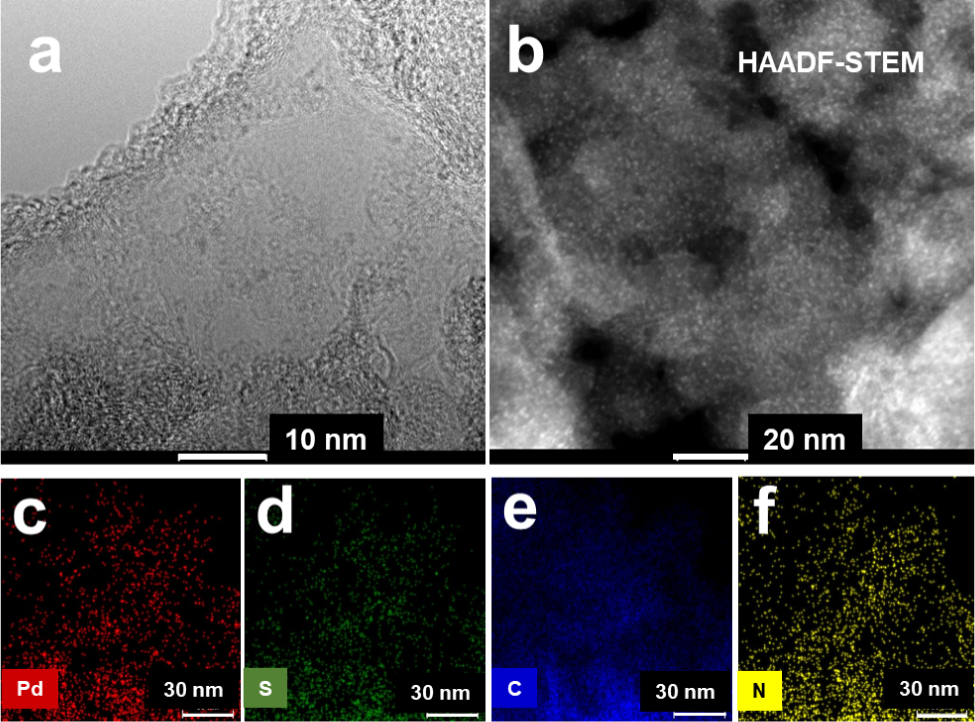
HRTEM (a) and HAADF-STEM (b) images of GNP-HL1-PdS; element distribution mapping for GNP-HL1-PdS (c: palladium, d: sulfur, e: carbon, and f: nitrogen).

The elemental composition data of the obtained GNP-HL1-PdS solid calculated from the corresponding survey XPS spectrum (Table S2) afforded the Pd/S = 1 atom relationship indicating that the whole of the palladium of the GNP-HL1-PdS sample was precipitated as PdS, thus discarding the existence of any Pd(0) in the precursor GNP-HL1-Pd.

The HR XPS spectrum of GNP-HL1-PdS in the Pd 3d region (Figure 3c) shows two signals at 337.6 and 342.8 eV, corresponding to 3 d_5/2_ and 3 d_3/2_ states. These BE values fit those of oxidized Pd^74^ but are slightly lower than those of the corresponding Pd­(II) precursor (338.0 and 343.2 eV, see above). This effect is similar to that observed in the case of the analogous G-Tren-PdS hybrid,^27^ and it is due to the different electronegativity of the N (amine) or S^2–^ donor atoms. The almost total disappearance of Cl signals in the XPS spectrum of the GNP-HL1-PdS hybrid (Table S2) is consistent with a quantitative Pd­(II) precipitation as PdS. Also, it supports the idea that in the GNP-HL1-Pd, chloride acts as an auxiliary ligand (as reasoned above in the light of [9]­aneN_3_ crystal structures).

The HR XPS spectrum in the N 1s region of GNP-HL1-PdS (Figure 3a) shows a main signal with two components at BE values similar to those in the GNP-HL1-Pd spectrum, which correspond to the triamine ring atoms (the one at the lower BE, ca. 399.2 eV) and to four of the five aromatic N atoms (the one at ca. 400.3 eV). This indicates that in this hybrid, the interaction of the triamine ring moiety of HL1 remains associated with PdS nanoparticles, which is important regarding their stabilization on the GNP surface. Moreover, another signal appears in N 1s region, at BE values of 405.2 eV, indicating that, similar to what observed in GNP-HL1-Pd hybrid (see above), C(5)­NO group of the pyrimidine (possessing a soft ligand character)^72,73^ also interacts with PdS NPs, as if NPs are somewhat built atop/rest upon the pyrimidine residues, further contributing to their stabilization. The larger shifting of this signal in the GNP-HL1-PdS spectrum than in the GNP-HL1-Pd one indicates that the interaction is stronger in the former. Accordingly, NPs are more interacting with the surface, while discrete Pd­(II) ions appear chiefly coordinated to the macrocycle.

The HR XPS spectrum of GNP-HL1-PdS in the S 2p (Figure 3e) region contains two components at 164.2 and 162.9 eV corresponding to 2 p_1/2_ and 2 p_3/2_ states. A weak wide peak at 168.0 eV is assigned to satellites of 1/2 and 3/2 states of PdS, which are related to transitions between the VB and CB of PdS NPs,^74,78^ thus confirming the semiconductor character of the precipitated PdS NPs.

The UV–visible spectrum of a water suspension of GNP-HL1-PdS is shown in Figure 2c. In this spectrum, the absorption in the 200–400 nm range comes from HL1 and from the graphene component as discussed above. On the other hand, the high absorbance in the whole visible comes from the sum of π→π* transitions of isolated sp^2^ domains in GNP-HL1 surface (with a maximum at ca. 1.5 eV, see above) and from the expected VB → CB (bandgap) transitions of the deposited PdS semiconductor NPs. According to the literature data^33,79–81^ and to the results obtained in a previous work when PdS NPs were deposited on G-Tren surface,^27^ the bandgap energy for PdS NPs on GNP-HL1 surface should be very close to that found for GNP-HL1 phase, namely 1.8 eV. This is consistent with the Tauc plot for direct transitions of Figure 2d, obtained from the visible spectrum of GNP-HL1-PdS. Figure 2d shows two overlapping straight segments with similar slopes in the ca. 3.6–4.2 eV range which should correspond to the above-mentioned bandgap transitions, which prevented accurate extrapolation to calculate the bandgap energy of PdS. Thus, it could be assumed that the bandgap of PdS is next to that of GNP-HL1 (ca. 1.8 eV), which is also similar to that previously calculated for G-Tren-PdS-CdS.^27^

#### 3.3.4 Preparation and Characterization of GNP-HL1-PdS-CdS

GNP-HL1-PdS-CdS hybrid was obtained by the precipitation of CdS under slow addition of equivalent amounts of Na_2_S and CdAc_2_ solutions, to a water suspension of the GNP-HL1-PdS precursor (Scheme 2 and see Supporting Information). The HAADF image of GNP-HL1-PdS-CdS (Figure 6a) shows a nonuniform distribution of crystalline aggregates spread on the surface of GNP-HL1, which correspond to CdS. The image also exhibits the presence of small PdS NPs, homogeneously distributed and stabilized by the nitrogen functions of GNP-HL1 surface. Deeper morphological analysis of GNP-HL1-PdS-CdS material was done from the HRTEM image of Figure 6h. This image evidences the existence in the GNP surface of the above material of lattice fringes with interplanar spacings of 0.336 and 0.206 nm, which can be assigned to the (111) and (220) crystallographic planes of cubic CdS (COD code 1011260; AMCSD code 0018121).^82–84^ The extremely low amount of PdS present in this material (<0.5 wt %) probably prevents observation in Figure 6h of any lattice fringe assignable to it.

**Figure 6:**
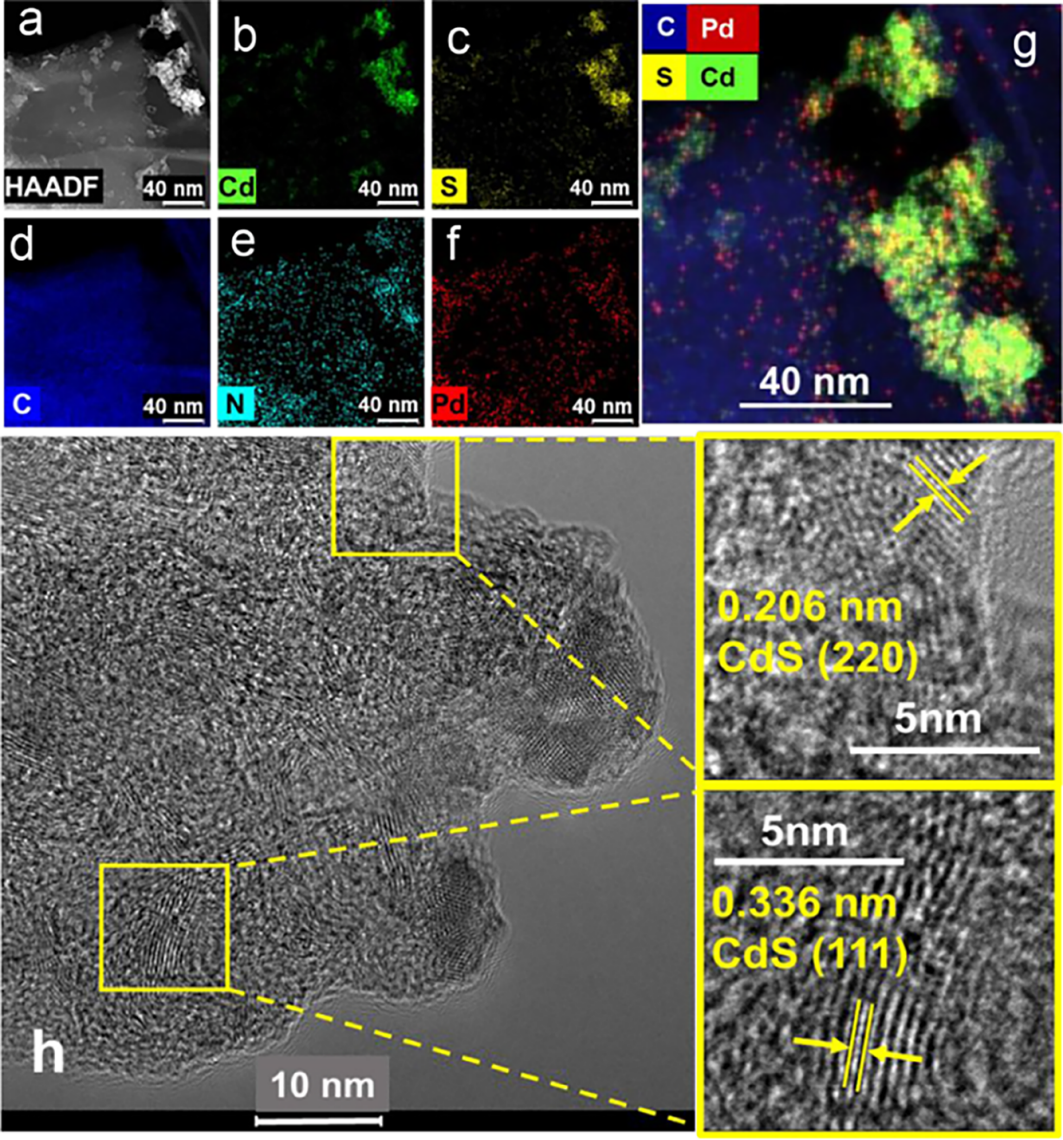
HAADF-STEM image of GNP-HL1-PdS-CdS (a); element distribution mapping of GNP-HL1-PdS-CdS: b) cadmium, c) sulfur, d) carbon, e) nitrogen, f) palladium, and g) all the elements; h) HRTEM image of GNP-HL1-PdS-CdS.

The element distribution mapping of GNP-HL1-PdS-CdS (Figure 6) illustrates the extended Csp^2^ moiety of GNP acting as support of both nitrogen (associated with HL1 molecules) and Pd (mostly as PdS) quite uniformly spread on the carbon support.

Different from the last, a similar nonuniform surface distribution pattern of both Cd and S is observed, which comes from the trend of CdS particles to aggregate to each other as was shown above (Figure 6a). In spite of this, it is seen in Figure 6g that most of both CdS and PdS particles keep close contact with each other, favoring the charge transfer between the two phases as required for efficient functioning of the photocatalyst.

In the XRD diffraction pattern of GNP-HL1-PdS-CdS (Figure S10), three peaks at 26.7°, 44.2°, and 52.0° are observed, which are assigned to (111), (220), and (311) planes of cubic CdS, respectively.^85^ The low content of both GNP (<5 wt %) and PdS (<0.5 wt %) components in the photocatalyst prevents detection of their characteristic diffraction peaks.

However, the HR XPS spectrum of GNP-HL1-PdS-CdS shows weak, well-defined signals in the Pd 3d region (Figure 7a) at similar BE values as in the spectrum of its GNP-HL1-PdS precursor. The C 1s signal also appears clearly defined in the spectrum of Figure 7b, and it is worth mentioning that the signal is significantly wider and shows a smoother maximum than that of the GNP-HL1-PdS precursor (Figure S11a). This probably comes from the CdS aggregates nonuniformly depositing on the GNP surface. A very weak signal of the main component of N 1s (Figure 7c, inset) appears at a similar BE value (ca. 399.0 eV) as in the GNP-HL1-PdS precursor (Figure S11b), although its contribution is mostly hidden by the signal assigned to Cd 3d_5/2_ (Figure 7c). In the Cd 3d region, another component, due to the 3d_3/2_ state of Cd, appears at 411.0 eV as a sharp signal.^74^ Finally, the intense signals with maxima at 160.5 eV and a shoulder at 161.6 eV (Figure 7d) should be due to 2p_3/2_ and 2p_1/2_ states of S, coming mainly from the major component, CdS, which hides the sulfur signal from the much minor component, PdS, which should nevertheless appear at similar BE values.

**Figure 7:**
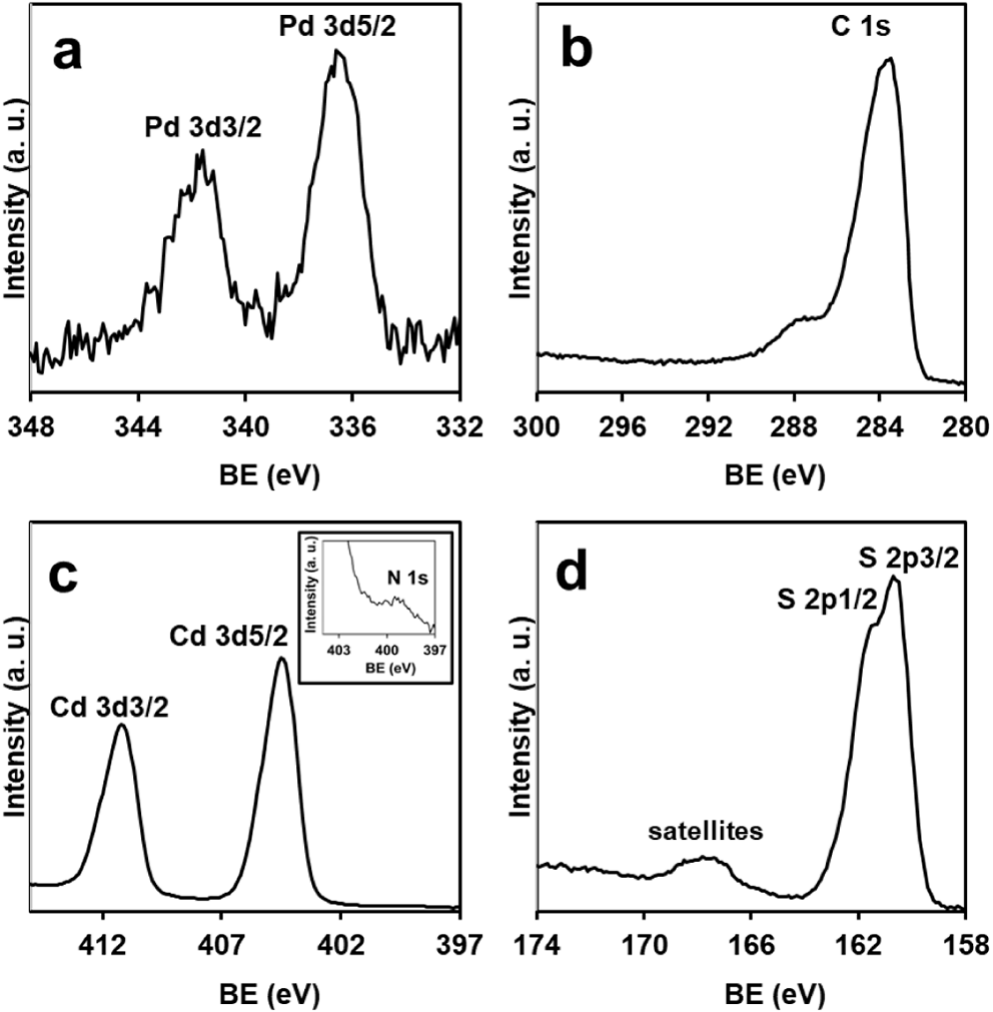
HR XPS of GNP-HL1-PdS-CdS in the a) Pd 3d, b) C 1s, c) Cd 3d (inset, N 1s), and d) S 2p regions.

The weak wide signal at ca. 167.5 eV is assignable to the satellites of the S components of CdS (hiding that of the minor component PdS) and is indicative of the semiconductor character of CdS NPs of GNP-HL1-PdS-CdS.

The UV–visible diffuse reflectance spectrum of GNP-HL1-PdS-CdS appears in Figure 2e. The broad absorbance of the hybrid in the 400–600 nm range should be due mainly to CdS^27^ (the main component, ca. 95 wt %). Although the GNP-HL1-PdS component also absorbs radiation in the above range, its contribution should not be significant as it is found in a small amount in the photocatalyst. On the contrary, the whole absorbance observed in the 600–800 nm range, in which the absorbance of CdS is very small, is ascribed to the GNP-HL1 component. The Tauc plot obtained from the UV–visible spectrum of G-HL1-PdS-CdS (Figure 2f) only shows a direct transition, at 2.5 eV. This value is in the range of those given in the literature for the direct bandgap transition of CdS, to which it is assigned.^16,27^ The low amounts of the other semiconductor phases present in the photocatalyst (GNP-HL1 and PdS) probably prevent the observation, in Figure 2f, of any other bandgap transition assignable to them.

#### 3.3.5 Photocatalytic Studies

In the photocatalytic studies, a SO_3_^2–^ + S^2–^ mixture was used as a a sacrificial agent instead of S^2–^, as in the last case photocorrosion of CdS and PdS is promoted due to the reaction product formed, S_2_^2–^, which competes with the protons in the reaction with the photogenerated electrons.^86^

By following the procedure described in Section 2.6, the GNP-HL1-PdS-CdS photocatalyst was irradiated for 28 h (Figure S12). This time fits that found in the literature for most of CdS-based photocatalysts.^87,88^ Figure S12 shows an almost straightforward hydrogen evolution vs time, which is consistent with the great stability of the photocatalyst. To shed light on the mechanism for the water reduction reaction catalyzed by GNP-HL1-PdS-CdS, the photocatalytic activities of this material up to 14 h and those of CdS, GNP-CdS, GNP-HL1-CdS, GNP-H_2_L2-CdS, PdS-CdS, and GNP-H_2_L2-PdS-CdS are summarized in Figure 8 for comparison. All materials tested have similar amounts of CdS and/or PdS and have been prepared following a similar experimental procedure.

**Figure 8:**
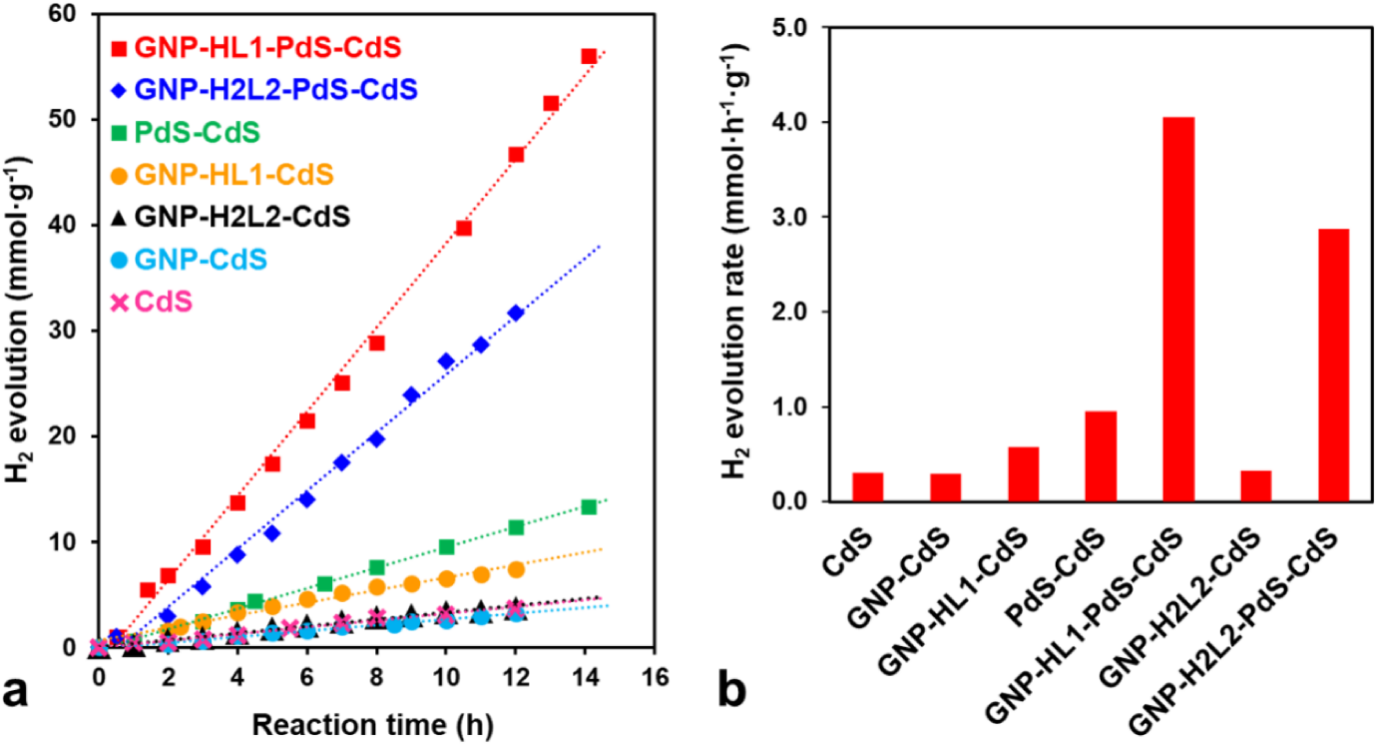
Photocatalytic activity in water reduction reaction under visible light irradiation, using 5·10^–3^ g of the corresponding photocatalyst in 0.35 M Na_2_S/0.25 M Na_2_SO_3_ mixed aqueous solution: a) hydrogen evolution for GNP-HL1-PdS-CdS, GNP-H_2_L2-PdS-CdS, PdS-CdS, GNP-HL1-CdS, GNP-H_2_L2-CdS, GNP-CdS, and CdS catalysts; b) comparison of the photocatalytic H_2_ production activity of all assayed catalysts.

GNP-HL1-PdS-CdS shows a great performance in the photocatalytic essays, yielding an average value of 4.05 mmol·g^–1^·h^–1^ over 14 h, which remained almost constant up to 28 h (Figure S12). This value is almost double that obtained in a previous work for the analogous G-Tren-PdS-CdS material with similar contents of PdS and CdS (which yielded an average value of 2.30 mmol of H_2_·g^–1^·h^–1^) when it was used as a photocatalyst under similar experimental conditions.^27^ In addition, the AQE value (%) of GNP-HL1-PdS-CdS was calculated as 17% at 420 nm. Furthermore, the data of Table 2 show that the hydrogen evolution rate of the photocatalyst prepared in this work is higher than that of other similar composites reported in the literature,^27,40,87–94^ making it an excellent photocatalyst for water reduction reaction.

**Table 2:** Comparison of the Photocatalytic Data of GNP-HL1-PdS-CdS and Analogous Graphene-CdS-Based Composites

Photocatalyst	Light source	Sacrificial Agent	Activity (mmol·h^–1^·g^–1^)	Reference
GNP-HL1-PdS-CdS (4.5 wt %/≤0.5 wt %/95 wt %)	Simulated solar irradiation (AM 1.5G)	Na_2_S/Na_2_SO_3_	4.05	This work
G-Tren/PdS/CdS (5 wt %/0.5 wt %/94.5 wt %)	Simulated solar irradiation (AM 1.5G)	Na_2_S/Na_2_SO_3_	2.30	^27^
g-C_3_N_4_/Pd/CdS (15 wt %/5 wt %/80 wt %)	300 W Xe lamp (λ ≥ 420 nm)	Na_2_S/Na_2_SO_3_	0.293	^40^
G-MoS_2_/CdS (2 wt %/98 wt %)	300 W Xe lamp (λ ≥ 420 nm)	Lactic Acid	1.80	^87^
G-MoS_2_/CdS (2 wt %/98 wt %)	300 W Xe lamp (λ ≥ 420 nm)	Na_2_S/Na_2_SO_3_	1.20	^87^
3DGN/Pt/CdS (2 wt %/1 wt %/97 wt %)	300 W Xe lamp (λ ≥ 400 nm)	Na_2_S/Na_2_SO_3_	2.310	^88^
GSw/Pd/CdS (4.5 wt %/0.5 wt %/95 wt %)	Simulated solar irradiation (AM 1.5G)	Na_2_S/Na_2_SO_3_	3.79	^89^
G-Nanoribbons/Pt/CdS (2 wt %/1 wt %/97 wt %)	300 W Xe lamp (λ ≥ 400 nm)	Lactic Acid	1.891	^90^
G-WS_2_/Pt/CdS (4.2 wt %/1 wt %/94.8 wt %)	500 W Xe lamp (λ ≥ 420 nm)	Na_2_S/Na_2_SO_3_	1.842	^91^
Bi doped-CQDs/CdS (1 wt %/99 wt %)	300 W Xe lamp (λ ≥ 420 nm)	Na_2_S/Na_2_SO_3_	1.77	^92^
g-C_3_N_4_/CuS/CdS (3 wt %/10 wt %/87 wt %)	350 W Xe lamp (λ ≥ 420 nm)	Na_2_S/Na_2_SO_3_	1.20	^93^
N doped-G/CdS (2 wt %/98 wt %)	300 W Xe lamp (λ ≥ 420 nm)	Na_2_S/Na_2_SO_3_	1.05	^94^

Analysis of the HR XPS in the Pd 3d region of fresh and used (for 28 h) GNP-HL1-PdS-CdS catalyst (Figures 7a and S13a) shows no significant reduction of Pd­(II) after using the catalyst. Moreover, neither significant changes of the signals of 2 p_1/2_ and 2 p_3/2_ states of S component nor of that at 404.3 and 411.0 eV corresponding to 3 d_5/2_ and 3 d_3/2_ states of Cd are observed (Figures 7 and S13). Although, as said before for the fresh photocatalyst, most of N 1s signal in the spectrum is hidden by that of the 3d_5/2_ component of Cd, a very weak signal due to the main component of N 1s appears in the XPS of the used catalyst at the same BE as that in case of the fresh one (Figures 7c and S13c, inset). Moreover, no release of HL1 in water media was observed after the photocatalytic reaction. Thus, alongside its very good performance, these facts let one assume that GNP-HL1-PdS-CdS photocatalyst displays good stability during water reduction reaction.

Relative positions of the more external energy levels of the different components of the photocatalyst (CdS and PdS semiconductors and GNP-HL1 matrix) are essential to get insight into the possible role played by each of them in the studied reaction. For this purpose, the VB and CB energies of CdS and PdS semiconductors can be calculated using their bandgap values and the Mulliken equation,^27,95^

1 $$E_{\mathrm{VB}}=X-E^{e}+0.5{\;E}_{g}$$

2 $$E_{\mathrm{CB}}=E_{\mathrm{VB}}-E_{g}$$

where E_VB_ and E_CB_ are the valence band and conduction band energies, respectively, E^e^ is the energy of free electrons on hydrogen scale with a fixed value of 4.5 eV vs NHE, X is the geometric mean of the electronegativity of the semiconductor, which is calculated from the ionization potential and the electronic affinities of the corresponding atomic components, and E_g_ is the bandgap of the semiconductor.

In the case of CdS, the value of E_g_ = 2.5 eV is obtained from the Tauc plot of Figure 2f, whereas in that of PdS, according to the analysis of the Tauc plot of GNP-HL1-PdS (see above), it could be taken a rough value of 1.8 eV, which is consistent with that found for the analogous G-Tren-PdS^27^ and for small PdS nanoparticles.^67,79–81^ The values obtained on applying equations (1) and (2) for CdS were E_VB_ = 1.9 eV and E_CB_ = −0.6 eV, and for PdS, E_VB_ = 1.8 eV and E_CB_ = 0.0 eV, all of them in a similar range to those obtained in the case of G-Tren-PdS-CdS.^27^ These values are aligned together in Scheme 3.

**Scheme 3:**
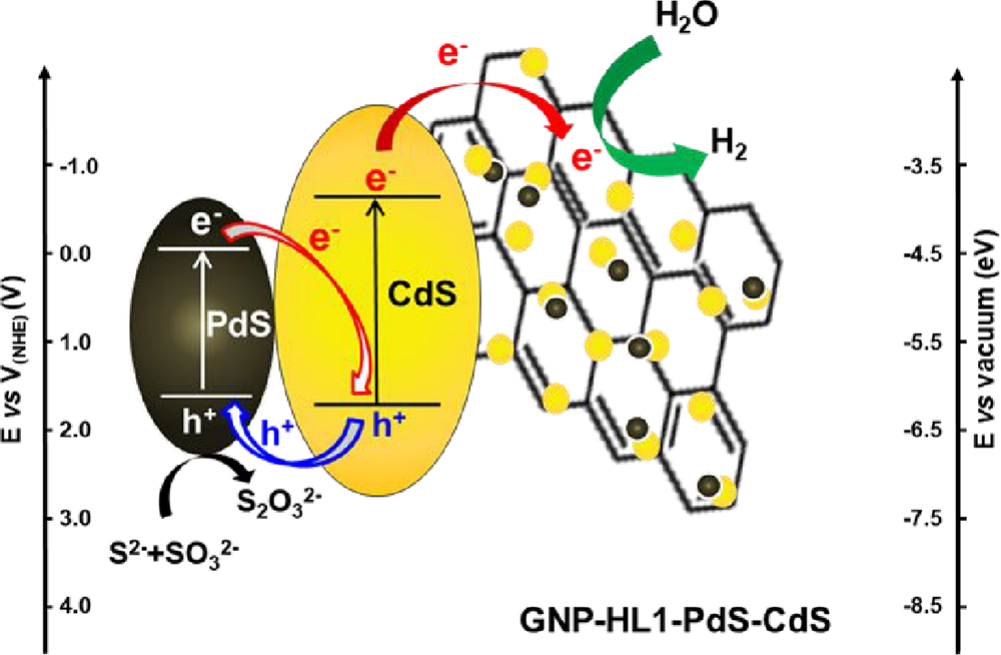
Schematic Illustration of the Proposed Mechanism for the Water Reduction Reaction Photocatalyzed by GNP-HL1-PdS-CdS

According to the literature, the work function of pristine graphene, ca. 4.3 eV, lies almost level with that of hydrogen in the vacuum scale^3,96^ and must increase on the attachment to the surface of withdrawing oxygen groups.^97^ Nevertheless, the increase should be modest if the oxygen content is less than 8 wt %,^98^ which is the case of GNP used in this work, so the work function of GNP should roughly level that of E^e^. However, in the basic medium of the photocatalytic reaction (pH ≥ 13, due to the hydrolysis of the sacrificial agent), the reduction potential of H_2_O/H_2_ pair shifts to a negative value of ca. −0.8 V relative to the standard (0.0 V).^32^ The splitting of VB and CB in GNP-HL1 when HL1 is adsorbed on GNP (see the above section) should result in setting the energy at the bottom of its (unoccupied) CB at a value suitable for the excited electrons to be able to reduce water. This hypothesis was assessed by analyzing HR-XPS spectra in the VB range of both GNP-HL1 and GNP. The HR-XPS spectrum of GNP-HL1 in the VB range (Figure S14) shows the edge of the maximum energy placed at about 1.1 eV (in the NHE scale). This value is more positive than the work function of the bare GNP, about 0.0 eV (Figure S14), as determined from its HR-XPS in the VB region (which is consistent with that stated above). On the other hand, as the bandgap of GNP-HL1 determined from the optical absorption spectrum is 1.8 eV, the minimum of the CB of GNP-HL1 would be set at about −0.7 eV in the NHE scale, almost leveling the hydrogen reduction potential (see above).^99^

Despite the above, neither GNP nor GNP-HL1 showed any observable photocatalytic activity under the experimental conditions used. This could be explained, in the case of the second, due to very quick charge recombination occurring into thin G sheets. The photocatalytic activity of GNP-CdS obtained by the slow precipitation of CdS on GNP matrix, 0.28 mmol·g^–1^·h^–1^, does not differ significantly from that of the bare CdS prepared by a similar procedure, 0.30 mmol·g^–1^·h^–1^, but increases significantly when using GNP-HL1-CdS, up to 0.57 mmol·g^–1^·h^–1^ (Figure 8). According to the above discussion, these results demonstrate that the splitting of G orbitals into VB and CB bands, i.e., the GNP-functionalization with HL1, makes the hydrogen reduction significantly more efficient. PL spectra of CdS and GNP-HL1-CdS are shown in Figure S15a. In spite of their low quality (see the Experimental Section), comparison of the spectra points out a clear decrease of the intensity of luminescence of GNP-HL1-CdS relative to that of CdS, especially in the range of the bandgap transition (ca. 520–570 nm), which strongly support transference of excited electrons from CdS to GNP-HL1. Thus, it can be assumed that electron transference to GNP-HL1 surface from the CB of the irradiated CdS, not only contributes to stabilize charge separation in CdS phase but GNP-HL1, together with CdS, most likely takes part in the water reduction (Scheme 3). It is worth mentioning also that the photocatalytic performance of GNP-HL1-CdS also exceeds, by a 2.3 factor, that of the analogous G-Tren-CdS tested in a previous work.^27^ Having in mind that compositions and optical properties of both hybrids are similar, the much better performance of GNP-HL1-CdS than G-Tren-CdS can be explained accounting for the reduced tendency toward stacking of sheets in the new material, as per the mentioned design strategies.

When GNP-HL1-PdS was used as a photocatalyst, no H_2_ production was observed. Assuming that the inactivity of GNP-HL1 in the photocatalytic reaction is due to quick electron–hole recombination (see above), the effectiveness of GNP-HL1-PdS in the photocatalytic reaction should be subject to suitable synergy of both GNP-HL1 and PdS. However, PdS is inactive in the photocatalytic reaction, which has been pointed out previously,^32^ since the excited electrons in the PdS phase (ca. 0.0 eV in NHE scale) are at less negative energy than the H_2_O/H_2_ potential reduction at the pH of the photocatalytic experiment (see above). Moreover, possible electron-positive hole transfer between PdS and GNP-HL1 phases, stabilizing charge separation in GNP-HL1, should be prevented, as the VB energy of holes in GNP-HL1 is more negative than the VB of PdS.

The photocatalytic activity of GNP-HL1-CdS (0.57 mmol·g^–1^·h^–1^ hydrogen production) greatly improves under a small loading of PdS (<0.5 wt %) up to 4.05 mmol mmol·g^–1^·h^–1^. It is worth mentioning that the hydrogen production with a PdS-CdS mixture (ca. 0.5 wt % of PdS) photocatalyst (0.77 mmol·g^–1^·h^–1^) also overcomes that of bare CdS by a factor of 2.6. Regarding this result, the PL spectrum of the PdS-CdS solid mixture used in this work (Figure S 15b) shows that the intensity of the emission in the whole 400–600 nm range (including the bandgap transition of CdS at 520–570 nm) decreases when compared to the bare CdS. This fact can be explained by the transference of excited electrons in the CB of PdS phase to positive holes in the VB of CdS, stabilizing charge separation in the last. This has been described in the literature for PdS-CdS photocatalysts^32^ used in similar conditions to those of this work, where it was also found that increasing PdS amounts above c.a. 0.1 wt % in the PdS-CdS mixture did not result in any enhancement of the H_2_ production.

When the PdS-CdS mixture is deposited on the GNP-HL1 matrix, the resulting GNP-HL1-PdS-CdS photocatalyst yields a photocatalytic H_2_ production (4.05 mmol·g^–1^·h^–1^) that increases that of the former by a 5.3 factor. This improvement is much higher than that found when a similar PdS-CdS mixture was deposited on the G-Tren matrix, to obtain G-Tren-PdS-CdS photocatalyst, which increased the PdS-CdS performance by a 3.0 factor.^27^ This result finally clarifies how low stacking of GNP-HL1 sheets (contrary to extended stacking in the G-Tren matrix) is the key to the improved photocatalytic performance, as the incident radiation can now more easily reach the catalytic and cocatalytic centers. The above, while maintaining the planar structure of the platform, favors efficient spreading of the electrons on its surface, so that they could promptly react with attached water species.^35^

Summarizing the above results, in the case of GNP-HL1-PdS-CdS, the GNP-HL1 matrix contributes to the improvement of the photocatalytic efficiency by (i) favoring spreading of both CdS NPs and PdS NPs, thus increasing their exposed surface area to the radiation; (ii) allowing the tuning of the CB energy of the matrix moiety, GNP-HL1 itself, thus enabling the electrons transferred from the CdS photocatalyst to actively take part in proton reduction, and (iii) stabilizing deposited metal NPs.

An additional question arises about the proton reduction on the graphene surface, which should rest on the nature of the interaction of graphene surface with water species.^34,35^

Regarding this issue, we prepared and studied the GNP-H_2_L2-PdS-CdS photocatalyst. This is analogous to GNP-HL1-PdS-CdS, with the key difference that the ligand contains 2 pyrimidine residues instead of one. The preparation was done as described above also for this system (see Supporting Information); i.e., a mixture of CdS and PdS nanoparticles was deposited on the GNP-H_2_L2 matrix (0.60 mmol of H_2_L2·g^–1^), resulting in similar relative amounts of the three (matrix, CdS, PdS) components. Functionalization of GNP with H_2_L2 (Scheme 2) results in GNP-H_2_L2, which behaves as a semiconductor with a bandgap of 2.3 eV assigned to a direct transition from VB to CB at c.a. 539 nm (Figure S16). This bandgap is wider with respect to that of GNP-HL1 (see above). Characterization of GNP-H_2_L2-PdS-CdS (Figure 9) also reveals a similar role of H_2_L2 molecules of the matrix surface in stabilizing CdS and PdS NPs as that of HL1 in the case of GNP-HL1-PdS-CdS.

**Figure 9:**
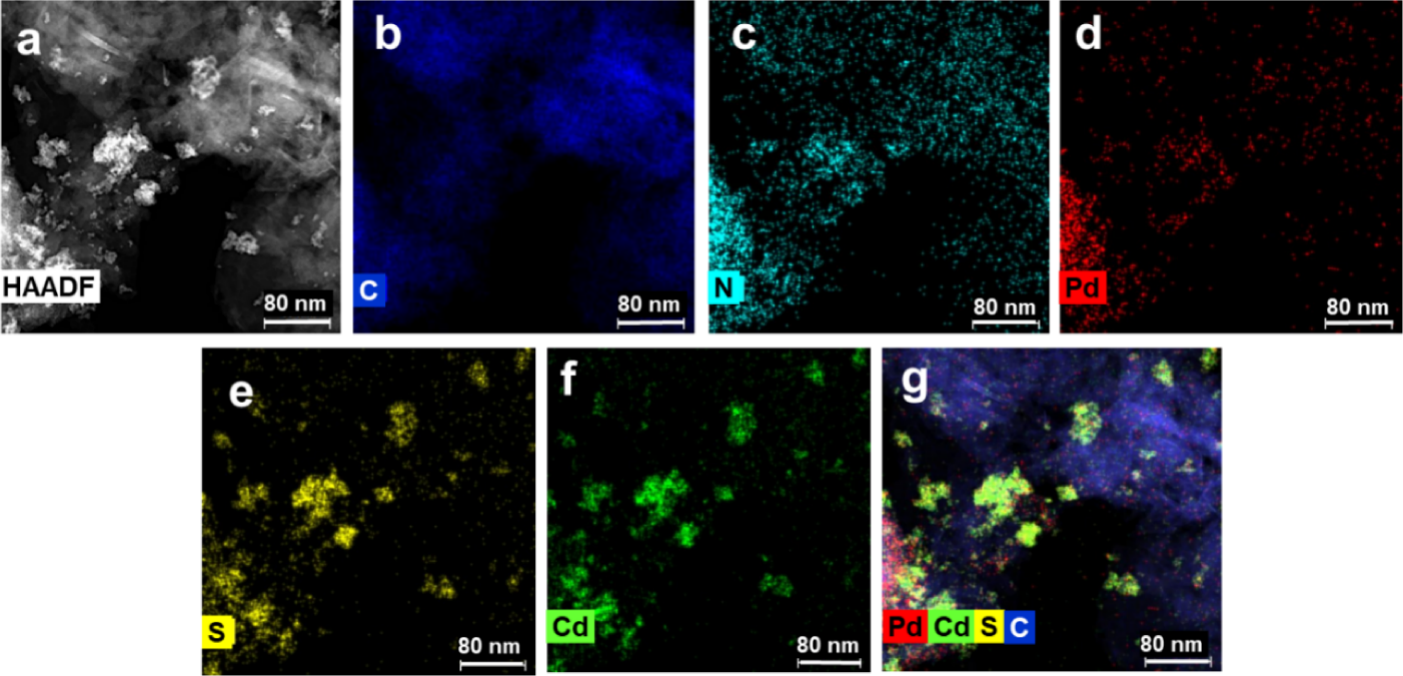
HAADF-STEM image of GNP-H_2_L2-PdS-CdS (a). Element distribution mapping of GNP- H_2_L2-PdS-CdS: b) carbon, c) nitrogen, d) palladium, e) sulfur, f) cadmium, and g) all the elements.

Different from the case of GNP-HL1-CdS (see above), the photocatalytic performance of GNP-H_2_L2-CdS (0.32 mmol·g^–1^·h^–1^, Figure 8) is practically not improved relative to the bare CdS (0.30 mmol·g^–1^·h^–1^). Moreover, the efficiency of photocatalytic H_2_ production when GNP-H_2_L2-PdS-CdS is used (2.88 mmol·g^–1^·h^–1^) is ca. 29 % smaller than that of the analogous GNP-HL1-PdS-CdS (4.05 mmol·g^–1^·h^–1^). Having in mind that the two photocatalysts show similarly low extension of stacking (Table S1), the much smaller efficiency of GNP-H_2_L2 matrix than GNP-HL1 in improving the photocatalytic performance of both CdS and PdS-CdS could be explained by structural differences between HL1 and H_2_L2. Assuming H_2_L2 (Scheme 1) is adsorbed via interaction of both pyrimidines on GNPs, the ligand causes more extended blocking of the Csp^2^ surface (as also supported by the determined bandgap).

The reduced photocatalytic performance demonstrates how water species need to interact with the composite catalysts via the Cπ centers of the matrix.^34–36^ This points out that, while partial blocking of the Cπ network is important to tune the bandgap of the matrix, excessive blocking of the sp^2^ system can be detrimental, as it constitutes the functional interface of the photocatalyst toward water species. Striking a balance between these two factors appears as a design goal to further improve the performance.

## 4 Conclusions

In this work, we addressed and identified some of the factors affecting the performance of PdS-CdS-based photocatalysts constructed on ligand-decorated graphene-based surfaces. The choice of GNP-HL1 platform/cocatalysts was determined by (i) the high electron 2D conductivity of pristine GNPs, (ii) their high specific surface area, despite reduced mean sheet diameter (with respect to G) and thus diminished interlayer stacking, and (iii) the possibility to adjust CB of GNP via supramolecular (π–π stacking) functionalization with HL1. This last fact allows GNP-HL1 to act as an acceptor for the light-promoted CdS electrons and as a donor toward H_2_O. The ligand-induced splitting of VB and CB is thus of paramount importance for the photocatalytic performance. Beyond bandgap arguments, the fact that water–composite photocatalyst interactions are mediated by Cπ centers was further corroborated by the study of the H_2_L2 analogue. Thus, we showed how a balance needs to be sought between sufficient (for bandgap purposes) and excessive (not enough water-accessible sites) blocking of the sp^2^ network characteristic of graphene-type surfaces. Reaching such a compromise involves the design of a suitable ligand for surface decoration.

The role of adsorbed ligand functionalities in both stabilizing and limiting the size of PdS nanoparticles deposited on the surface is also worth mentioning. As PdS is both expensive and the minor component of the photocatalyst (<0.5 wt %), limiting the size of these NPs is important for optimizing their contact with CdS, to maximize the light absorption ability and the lifespan of electron–hole pairs of the whole system and to contain costs.

The results of this work also point out that the textural features of the photocatalyst are of high importance for its performance. The reduced size of the graphene sheets of GNP compared to G and the suitable designing of the function adsorbed on GNP surface are key factors in order to limit the trend of graphene sheets to stack, which, as previously observed, can prevent light penetration to the inner (and minor, like PdS) components of the photocatalyst.^27^

Last but not least, when the above attention and strategies are simultaneously accounted for and implemented in a CdS-based system, we demonstrated in practice that they do lead to significantly improved performances with respect to state-of-the-art photocatalysts.

## Supplementary Material



## Notes

Preparation details for all studied catalysts and further figures, diagrams, and tables containing full characterization of discussed systems (PDF)
