# Supplementary material for: Supramolecular Assembly of Graphene–Polyamine–PdS–CdS Photocatalysts for Synergistically Enhanced and Highly Effective Hydrogen Evolution from Water under Visible Light
Source: Inorg Chem. 2025 Oct 7;64(41):20625–42. doi: 10.1021/acs.inorgchem.5c02898 (PMC12541699; doi:10.1021/acs.inorgchem.5c02898)
Supplement: Supplementary file 1 [file ic5c02898_si_001.pdf]

Supporting Information for

Supramolecular Assembly of Graphene-Polyamine-PdS-CdS Photocatalysts for Synergistically Enhanced and Highly Effective Hydrogen Evolution from Water under Visible Light.

María L. Godino-Salido,[a*] Francesco Bartoli,[b†] Alba M. Valbuena-Rus,[a†] Giuseppe Vicidomini,[c†] María D. Gutiérrez-Valero,[a] Victor K. Abdelkader-Fernández,[d] Matteo Savastano,[e,f] Antonio Bianchi,[c,f] Rafael López-Garzón[a]

[a] *Department of Inorganic and Organic Chemistry, Faculty of Experimental Sciences, Jaén University, 23071, Jaén, Spain. mlgodino@ujaen.es.*
[b] *Institute of Chemistry of Organometallic Compounds-National Research Council of Italy, Via Madonna del Piano 10, 50019 Sesto Fiorentino (Florence), Italy.*
[c] *Department of Chemistry "Ugo Schiff", University of Florence, Sesto Fiorentino, 50019, Florence, Italy.*
[d] *Department of Inorganic Chemistry, Faculty of Sciences, Granada University, 18071, Granada, Spain.*
[e] *Department for the Promotion of Human Science and Quality of Life, University San Raffaele Roma, 00166, Rome, Italy. matteo.savastano@uniroma5.it.*
[f] *National Interuniversity Consortium of Materials Science and Technology (INSTM), Research Unit of Florence, Via G. Giusti 9, 50121 Florence, Italy.*
† These authors *contributed equally*

Contents

Preparation of the photocatalysts and their precursors 3

Preparation of GNP-HL1 and GNP-H_2L2 3

Preparation of GNP-HL1-Pd and GNP-H_2L2-Pd 3

Preparation of GNP-HL1-PdS and GNP-H_2L2-PdS 3

Preparation of GNP-HL1-PdS-CdS and GNP-H_2L2-PdS-CdS 4

Preparation of CdS 4

Preparation of PdS-CdS 4

Preparation of GNP-HL1-CdS and GNP-H_2L2-CdS 4

Tables and Figures 5

Figure S1. Top: UV-Vis spectra of the HL1 ligand at different pH. Bottom: pH dependence of the 328 nm and 262 nm maxima superimposed to the species distribution diagram calculated for the ligand ([HL1] = $5.0 \cdot 10^{-5}$ M) according to the equilibrium constants in Table 1. 5

Figure S2. Top: UV-Vis spectra of the H_2L2 ligand at different pH. Bottom: pH dependence of the 326 nm and 267 nm maxima superimposed to the species distribution diagram calculated for the ligand ([H_2L2] = $2.0 \cdot 10^{-5}$ M) according to the equilibrium constants in Table 1. 6

Figure S3. Distribution diagrams for the HL1 (top) and H_2L2 (bottom) ligands in the presence of 1 eq of Cu(II) (left) or Cd(II) (right) (1 mM). Dashed red line: free metal ion. In some species-crowded areas, dash dot violet lines represent protonated free ligand forms. Charges omitted for simplicity. 7

Figure S4. Top left. UV-Vis spectra of a 1:1 solution of HL1 and Pd(II) at different pH ($5.0 \cdot 10^{-5}$ M). Bottom left: comparison of the pH dependence of the 328 nm band in the absence and in the presence of Pd(II). Top right. UV-Vis spectra of a 1:1 solution of H_2L2 and Pd(II) at different pH ($2.0 \cdot 10^{-5}$ M). Bottom left: comparison of the pH dependence of the 326 nm band in the absence and in the presence of Pd(II). ...8

Figure S5. a) Adsorption and desorption isotherms of HL1 on GNP; b) Pd(II) amounts adsorbed on GNP, GNP-HL1 and GNP-H_2L2. 8

Figure S6. XRD diffraction patterns of GNP, GNP-HL1, GNP-H_2L2 and GNP-HL1-Pd. 9

Table S1. Average values of the sizes packed units existing in GNP, GNP-HL1 and GNP-H2L2 materials calculated using the Scherrer equation* 9

Figure S7. N_2 adsorption and desorption isotherms of GNP and GNP-HL1 10

Figure S8. CO_2 adsorption and desorption isotherms of GNP and GNP-HL1 10

Figure S9. Plots of: a) $(\alpha E)^2$ (direct transitions) and b) $(\alpha E)^{1/2}$ (indirect transitions) vs the photon energy (E) for GNP; c) $(\alpha E)^{1/2}$ (indirect transitions) vs the photon energy (E) for GNP-HL1. 10

Table S2. Chemical composition (atomic concentration %) of GNP-HL1-PdS determined from the corresponding XPS survey spectrum 11

Figure S10. XRD pattern of GNP-HL1-PdS-CdS 11

Figure S11. HR XPS of GNP-HL1-PdS in the: a) C 1s and b) N 1s regions 11

Figure S12. Photocatalytic hydrogen evolution from 0.35 M Na_2S and 0.25 M Na_2SO_3 aqueous solution, using $5 \cdot 10^{-3}$ g of the catalyst GNP-HL1-PdS-CdS, under visible light irradiation. 12

Figure S13. HR XPS of irradiated GNP-HL1-PdS-CdS for 28 h in the: a) Pd 3d, b) C 1s, c) Cd 3d (inset N1s) and d) S 2p regions 12

Figure S14. XPS spectra in the VB region and values of the work functions of GNP-HL1 and GNP 13

Figure S15. PL spectra ($\lambda_{excitation}$: 425 nm) of: a) CdS and GNP-HL1-CdS, b) CdS and PdS-CdS 13

Figure S16. a) UV absorption spectrum of GNP-H_2L2-PdS-CdS; b) Plots of $(\alpha E)^2$ (direct transitions) vs the photon energy (E) for GNP-H_2L2-PdS-CdS. 14

Preparation of the photocatalysts and their precursors

Preparation of GNP-HL1 and GNP-H_2L2

GNP-HL1 hybrid material was prepared by adsorption of HL1 on GNP. The experimental conditions were selected by preliminary analysis of the corresponding adsorption and desorption isotherms obtained at 298.1 K in aqueous medium (Figure S5a), in order to obtain the maximum load of HL1 on GNP. Briefly, 0.100 g of the carbon support was suspended in 400 cm^3 of a $7.5 \cdot 10^{-4}$ M aqueous HL1 solution. The pH of the solution was adjusted to 5.0 by adding suitable amounts of 0.1 M HCl. The mixture (thermostated at 298.1 K) was stirred until the adsorption equilibrium was reached (48 h). The solid phase was separated by filtration, washed with distilled water and air dried. Then, it was left in water until desorption equilibrium of GNP-HL1 was reached. Finally, the resulting solid (containing 0.77 mmol of HL1$\cdot g^{-1}$) was separated by filtration, washed with distilled water and air dried again.

To obtain the GNP-H_2L2 hybrid material (containing 0.60 mmol of $H_2L2 \cdot g^{-1}$), a similar procedure was followed.

GNP-HL1 and GNP-H_2L2 were used as starting materials for the preparation of GNP-HL1-CdS, GNP-HL1-PdS-CdS, GNP-H_2L2-CdS and GNP-H_2L2-PdS-CdS photocatalysts.

Preparation of GNP-HL1-Pd and GNP-H_2L2-Pd

Once the GNP-HL1 and GNP-H_2L2 hybrids were obtained, GNP-HL1-Pd and GNP-H_2L2-Pd composites were prepared by suspending 0.1000 g of the hybrid in 200 mL of a 1 M KCl and 10^{-3} M K_2PdCl_4 aqueous solution, adjusting the pH at 5.0 by adding HCl 0.1 M. The corresponding mixtures were shaken at 298.1 K during 48 h, then the solids were separated by filtration, washed with distilled water and air dried. The amount of Pd(II) in the resulting materials (GNP-HL1-Pd 0.60 mmol$\cdot g^{-1}$ and GNP-H_2L2-Pd 0.61 mmol-H_2L2-Pd), was determined as the difference between the initial and equilibrium $PdCl_4^{2-}$ concentrations measured spectrophotometrically (at 474 nm). These values clearly exceed the amount of Pd(II) retained (under the same experimental conditions) on GNP (Figure 5b).

Preparation of GNP-HL1-PdS and GNP-H_2L2-PdS

The GNP-HL1-PdS and GNP-H_2L2-PdS materials were obtained by addition, drop by drop and under stirring, of an aqueous solution of 0.017 M Na_2S to an aqueous suspension of GNP-HL1-Pd or GNP-H_2L2-Pd (5 mg in 50 mL of water), up to the stoichiometric amount with respect to Pd(II). The resulting solids were separated by filtration, washed with distilled water and air dried. The Pd contents of these materials (6.5 wt% in the case of GNP-HL1-PdS and 6.7 wt% in case of GNP-H_2L2-PdS)

were determined as follows: c.a. 25 mg of the solid were mixed with 10 mL of a mixture of H_2SO_4/HNO_3 (2/1 v/v). The resulting suspension was heated under reflux up to the total solution of the sample. The solution was slowly evaporated up to dryness and the solid residue was solved in 2 mL of a HCl/HNO_3 (3/1 v/v) solution. Then, the Pd content of the solution was determined by ICP-MS using an AGILENT mod. series 7500 equipment.

Preparation of GNP-HL1-PdS-CdS and GNP-H$_2$L2-PdS-CdS

The GNP-HL1-PdS-CdS and GNP-H_2L2-PdS-CdS materials were obtained by slow CdS deposition on GNP-HL1-PdS and GNP-H_2L2-PdS, respectively. This was done by alternative addition to an aqueous suspension of GNP-HL1-PdS or GNP-H_2L2-PdS (5 mg in 5 mL of water) of small volumes (0.25 mL) of 0.025 M $Cd(CH_3COO)_2$ and Na_2S aqueous solutions, with continuous stirring, at 5 min time intervals between consecutive additions. The alternative addition prosecuted until the final amount of precipitated CdS was 95 wt%. The mixtures were stirred for 24 h and then centrifuged. The resulting solids, GNP-HL1-PdS-CdS and GNP-H_2L2-PdS-CdS, were recovered by filtration, washed with distilled water and ethanol, and dried at 100ºC for 24h. The Pd contents of these materials (0.33 wt% in the case of GNP-HL1-PdS-CdS and 0.37 wt% in case of GNP-H_2L2-PdS-CdS) were determined by ICP-MS as it has been described previously for GNP-HL1-PdS and GNP-H_2L2-PdS.

Preparation of CdS

CdS solid was also obtained by mixing, alternatively, small volumes of $Cd(CH_3COO)_2$ and Na_2S aqueous solutions, following the same precipitation procedure than that above described for the obtaining of GNP-HL1-PdS-CdS and GNP-H_2L2-PdS-CdS photocatalysts, but in absence of GNP-HL1-PdS/GNP-H_2L2-PdS precursors.

Preparation of PdS-CdS

A mixture of PdS and CdS, (PdS-CdS hereinafter), having similar composition (in terms of PdS/CdS ratio) than that of GNP-HL1-PdS-CdS, was prepared. For this, a PdS solid was first obtained by addition, drop by drop and under stirring, of an aqueous solution of 0.017 M Na_2S to an aqueous solution of K_2PdCl_4 (2.7 mg in 1 mL), up to stoichiometric amount regarding to Pd(II). Then CdS was slowly precipitated on the obtained PdS, following the same procedure described for the preparation of the GNP-HL1-PdS-CdS and GNP-H_2L2-PdS-CdS photocatalysts.

Preparation of GNP-HL1-CdS and GNP-H$_2$L2-CdS

GNP-HL1-CdS and GNP-H_2L2-CdS composites (with 95 wt % of CdS) were prepared by direct deposition of controlled amount of CdS NPs on the GNP- HL1 or GNP-H_2L2 hybrids, using the already described CdS precipitation procedure.

Tables and Figures

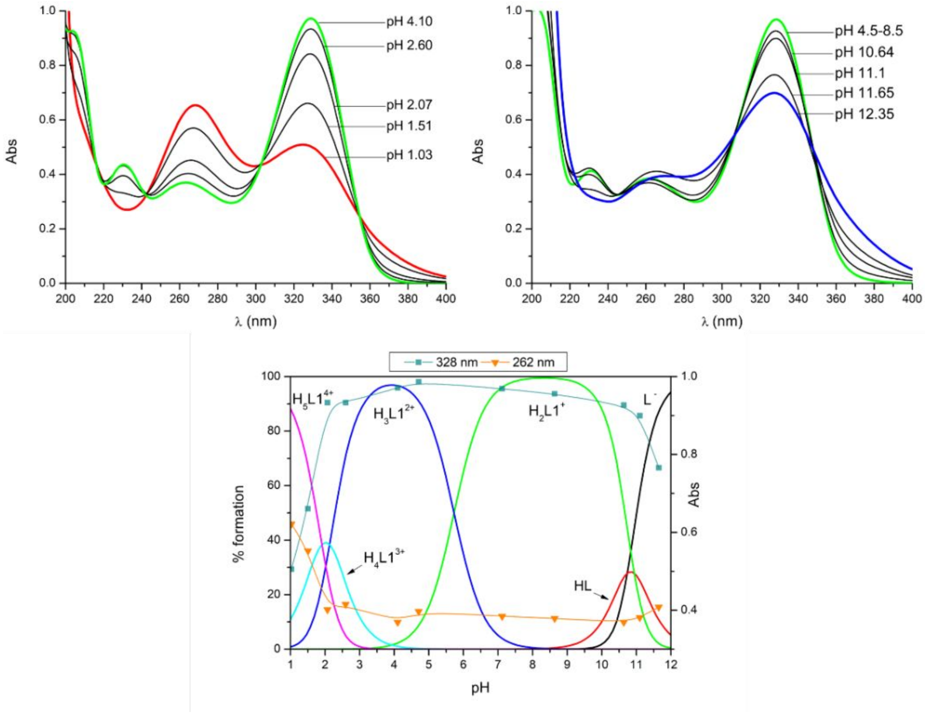

Figure S1. Top: UV-Vis spectra of the HL1 ligand at different pH. Bottom: pH dependence of the 328 nm and 262 nm maxima superimposed to the species distribution diagram calculated for the ligand ([HL1] = $5.0 \cdot 10^{-5}$ M) according to the equilibrium constants in Table 1.

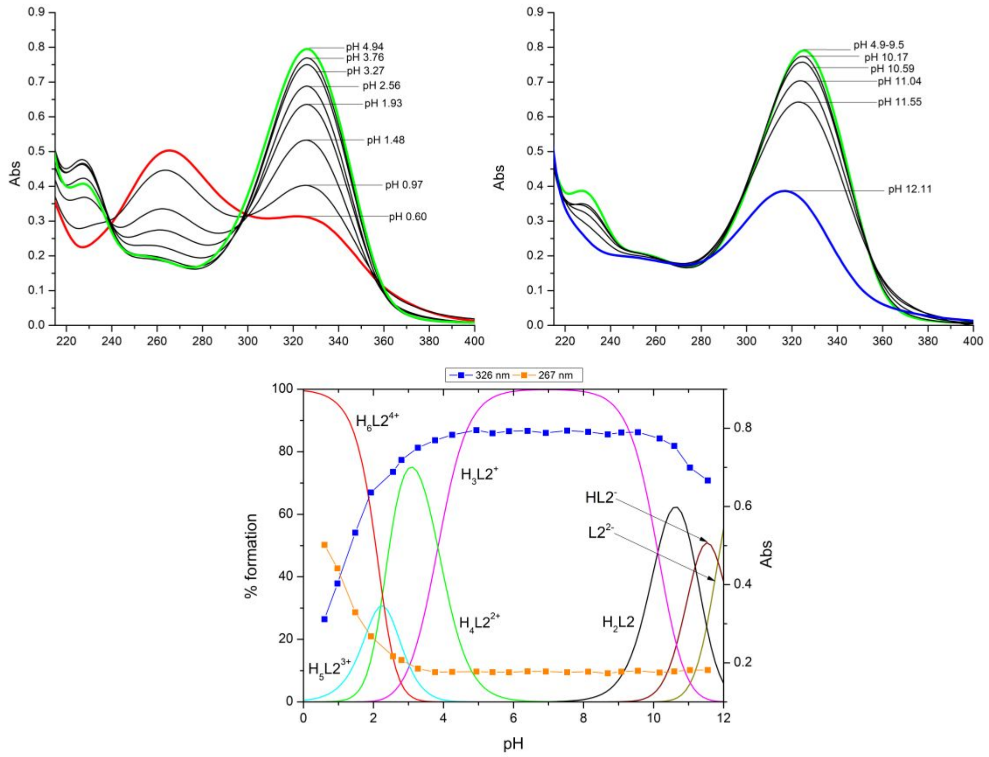

Figure S2. Top: UV-Vis spectra of the H_2L2 ligand at different pH. Bottom: pH dependence of the 326 nm and 267 nm maxima superimposed to the species distribution diagram calculated for the ligand ($[H_2L2] = 2.0 \cdot 10^{-5}$ M) according to the equilibrium constants in Table 1.

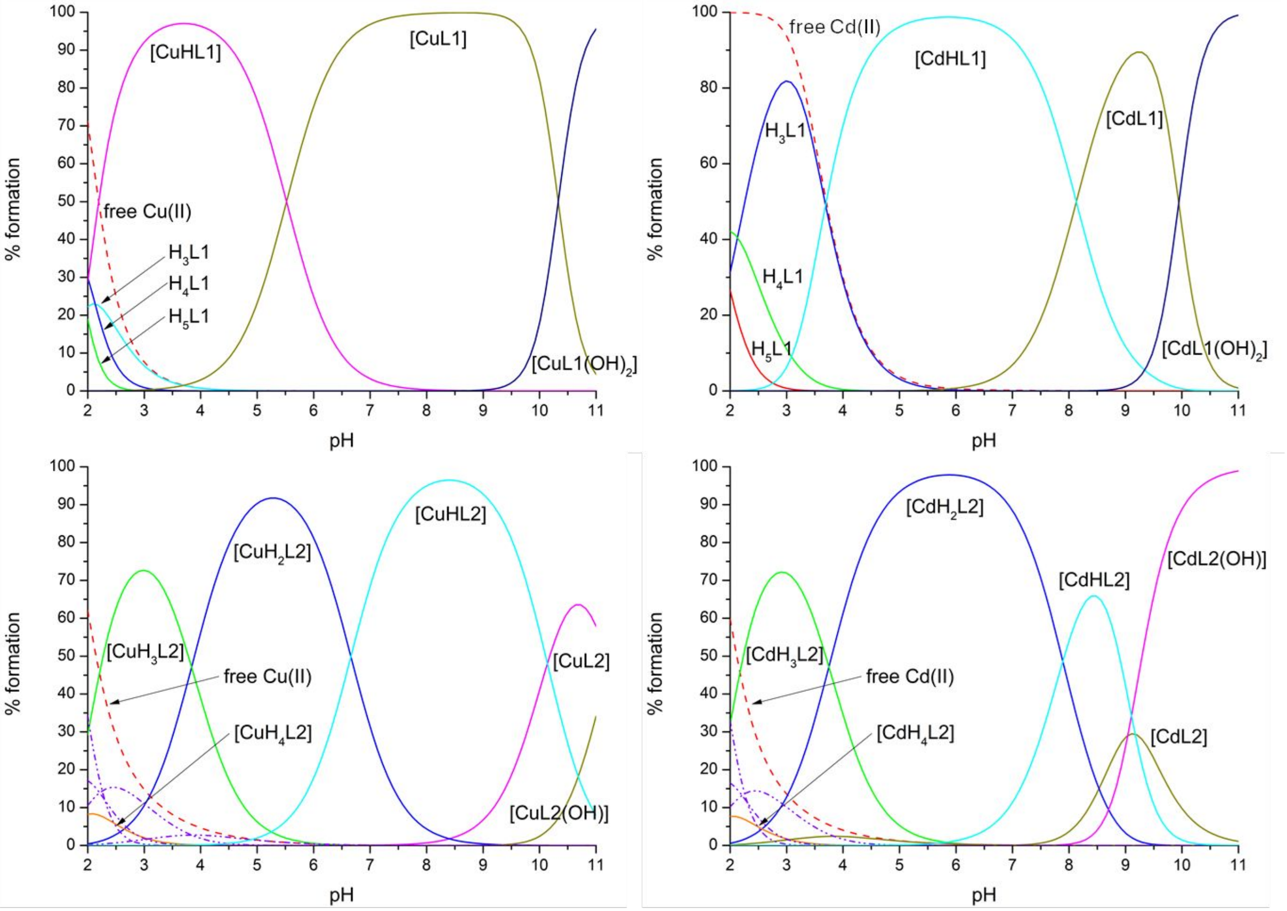

Figure S3. Distribution diagrams for the HL1 (top) and H_2L2 (bottom) ligands in the presence of 1 eq of Cu(II) (left) or Cd(II) (right) (1 mM). Dashed red line: free metal ion. In some species-crowded areas, dash dot violet lines represent protonated free ligand forms. Charges omitted for simplicity.

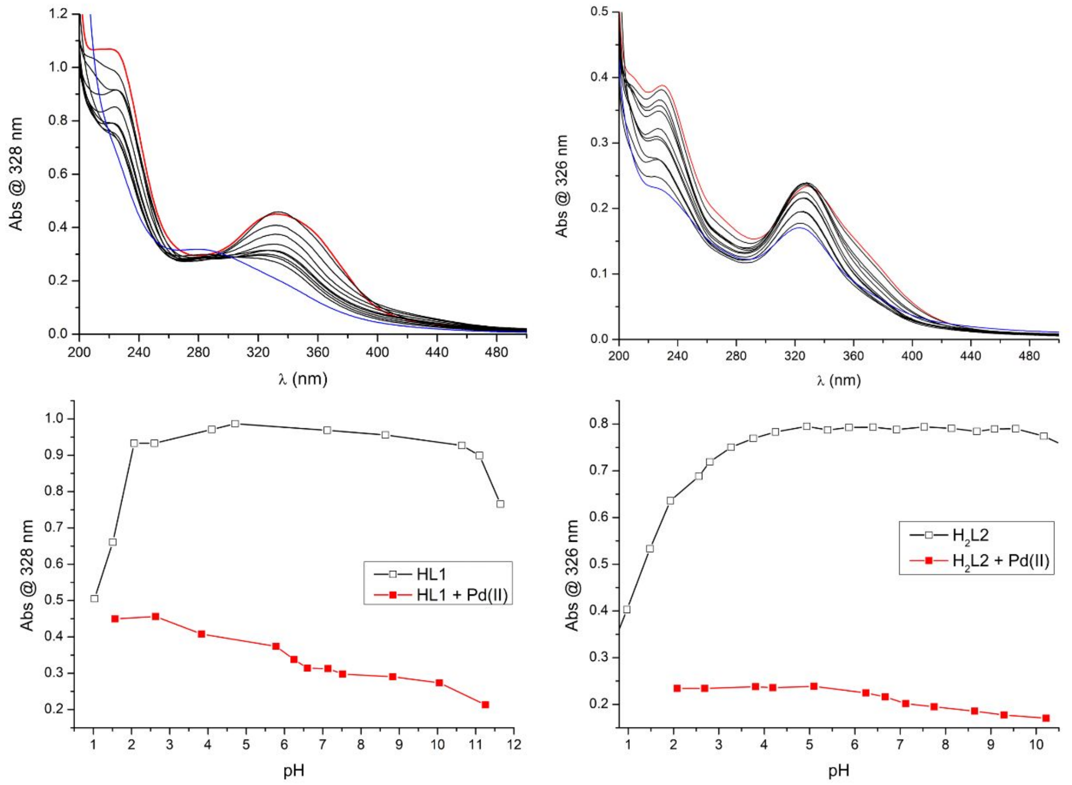

Figure S4. Top left. UV-Vis spectra of a 1:1 solution of HL1 and Pd(II) at different pH ($5.0 \cdot 10^{-5}$ M). Bottom left: comparison of the pH dependence of the 328 nm band in the absence and in the presence of Pd(II). Top right. UV-Vis spectra of a 1:1 solution of H_2L2 and Pd(II) at different pH ($2.0 \cdot 10^{-5}$ M). Bottom left: comparison of the pH dependence of the 326 nm band in the absence and in the presence of Pd(II).

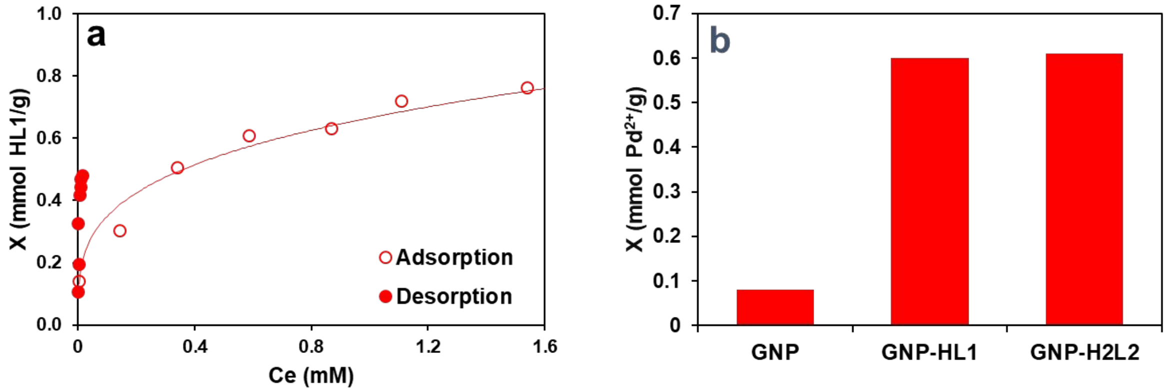

Figure S5. a) Adsorption and desorption isotherms of HL1 on GNP; b) Pd(II) amounts adsorbed on GNP, GNP-HL1 and GNP-H_2L2.

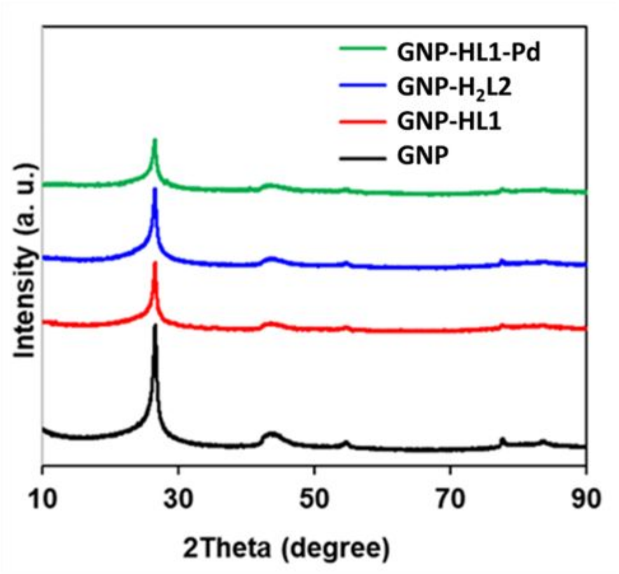

Figure S6. XRD diffraction patterns of GNP, GNP-HL1, GNP-H_2L2 and GNP-HL1-Pd.

Table S1. Average values of the sizes packed units existing in GNP, GNP-HL1 and GNP-H2L2 materials calculated using the Scherrer equation*

MATERIAL	2θ (º)	FWHM	D (nm)
GNP	26.5	0.962	8.63
GNP-HL1	26.5	1.050	8.12
GNP-H_2L2	26.5	1.213	7.03
GNP-HL1-Pd	26.5	1.981	4.30

*Scherrer Equation: $D = \frac{K\lambda}{\beta cos\theta}$

where D = size of the packed units (or crystals), K = Scherrer constant, λ = radiation's wavelength, β = FWHM (full width at half maximum), θ = diffraction angle.

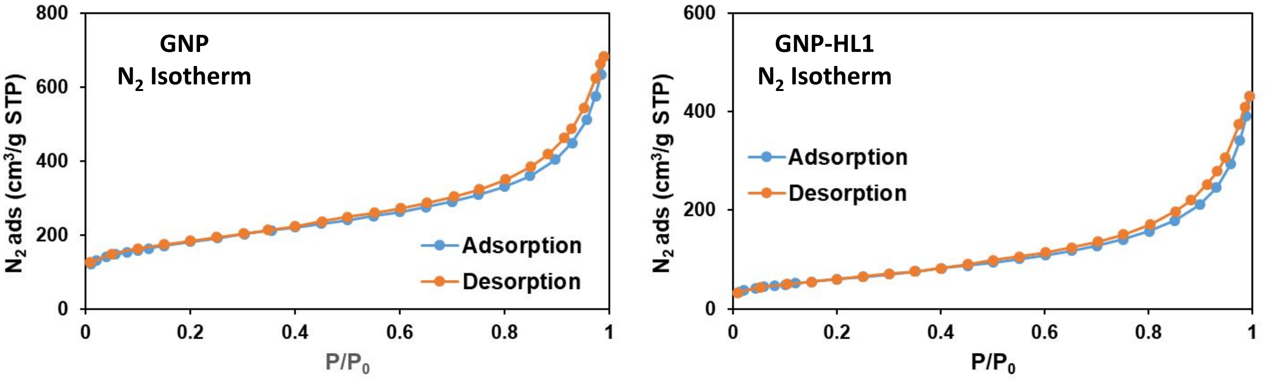

Figure S7. N_2 adsorption and desorption isotherms of GNP and GNP-HL1.

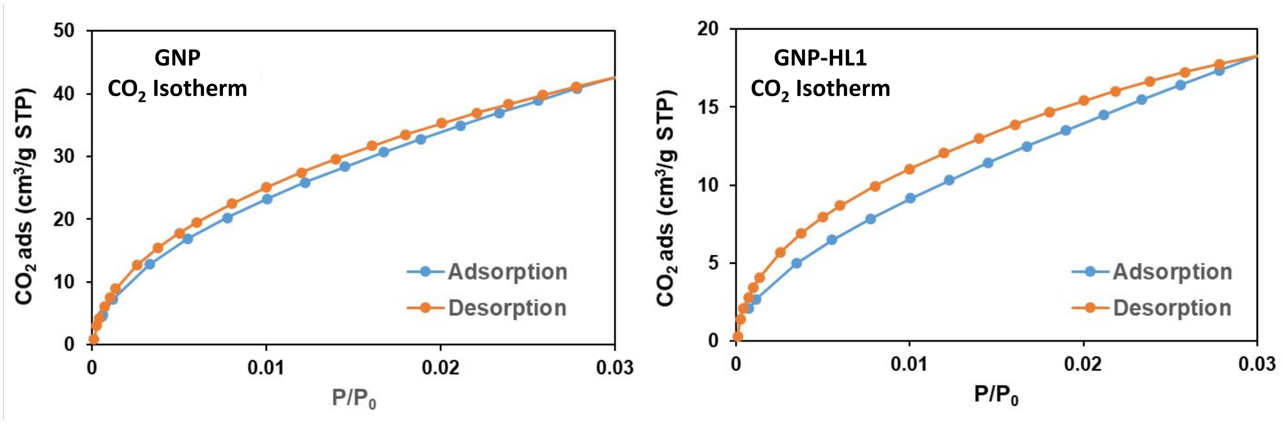

Figure S8. CO_2 adsorption and desorption isotherms of GNP and GNP-HL1.

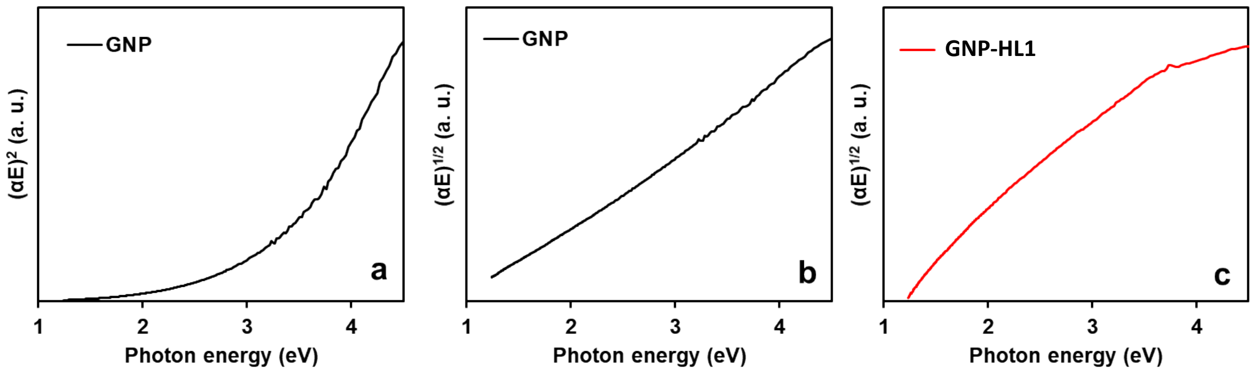

Figure S9. Plots of: a) $(\alpha E)^2$ (direct transitions) and b) $(\alpha E)^{1/2}$ (indirect transitions) vs the photon energy (E) for GNP; c) $(\alpha E)^{1/2}$ (indirect transitions) vs the photon energy (E) for GNP-HL1.

Table S2. Chemical composition (atomic concentration %) of GNP-HL1-PdS determined from the corresponding XPS survey spectrum.

	GNP-HL1-Pd	GNP-HL1-PdS
**Element**	**at%**	**at%**
C	83.46	78.56
O	10.69	16.85
N	4.41	3.55
Pd	0.57	0.48
S	-	0.48
Cl	0.87	0.08

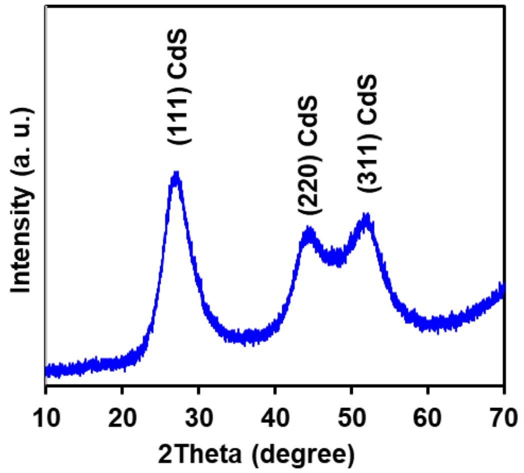

Figure S10. XRD pattern of GNP-HL1-PdS-CdS.

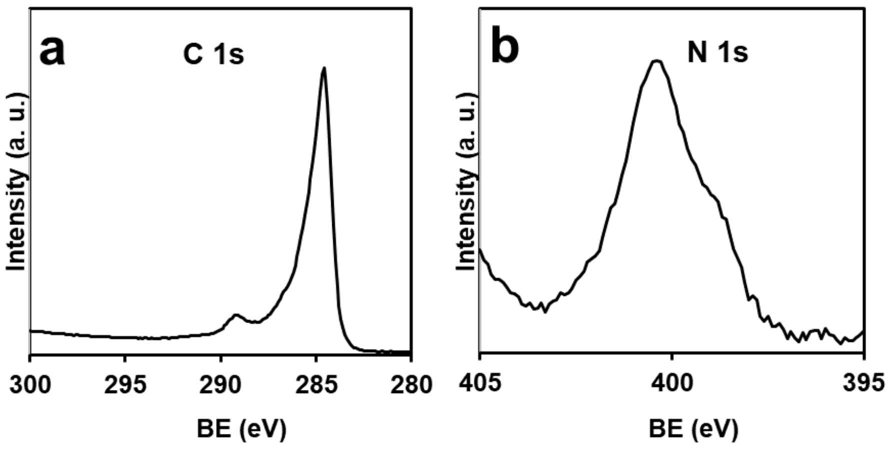

Figure S11. HR XPS of GNP-HL1-PdS in the: a) C 1s and b) N 1s regions.

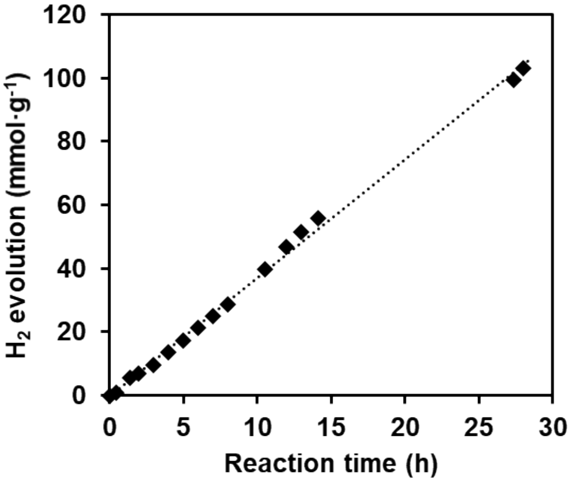

Figure S12. Photocatalytic hydrogen evolution from 0.35 M Na_2S and 0.25 M Na_2SO_3 aqueous solution, using $5\cdot10^{-3}$ g of the catalyst GNP-HL1-PdS-CdS, under visible light irradiation.

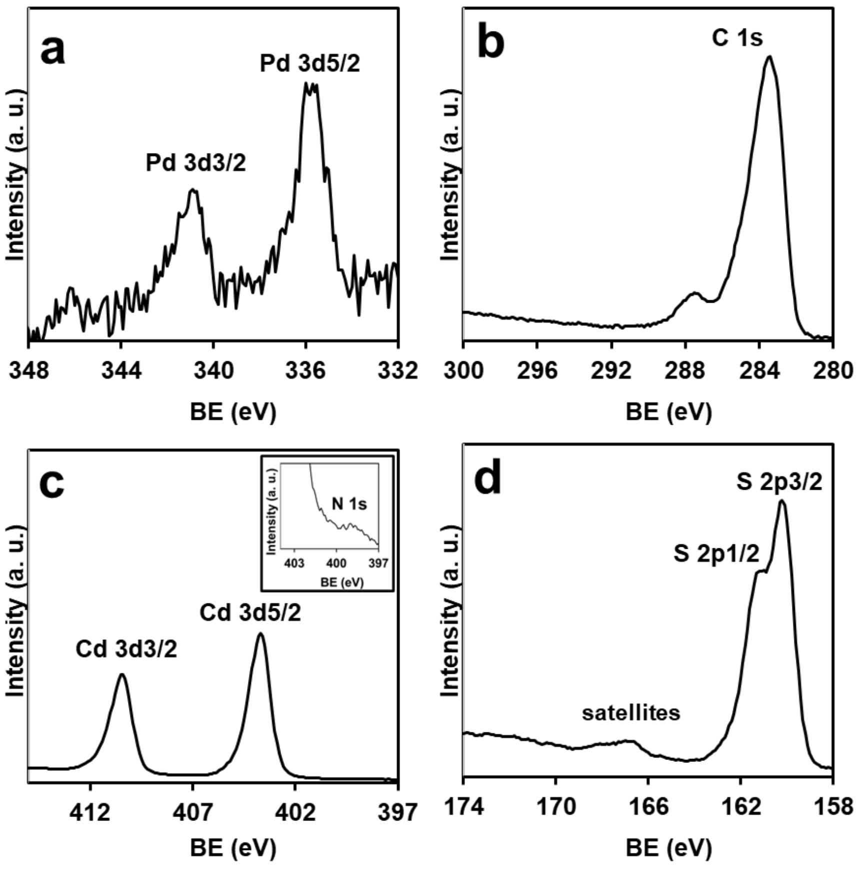

Figure S13. HR XPS of irradiated GNP-HL1-PdS-CdS for 28 h in the: a) Pd 3d, b) C 1s, c) Cd 3d (inset N1s) and d) S 2p regions.

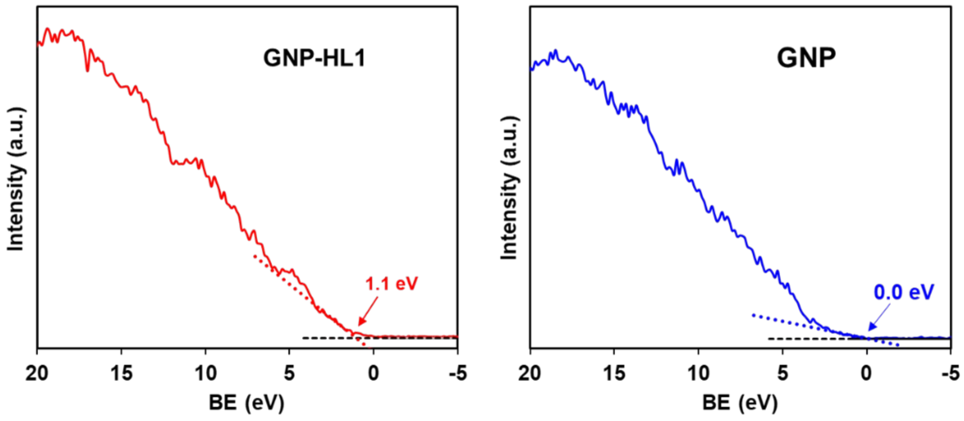

Figure S14. XPS spectra in the VB region and values of the work functions of GNP-HL1 and GNP.

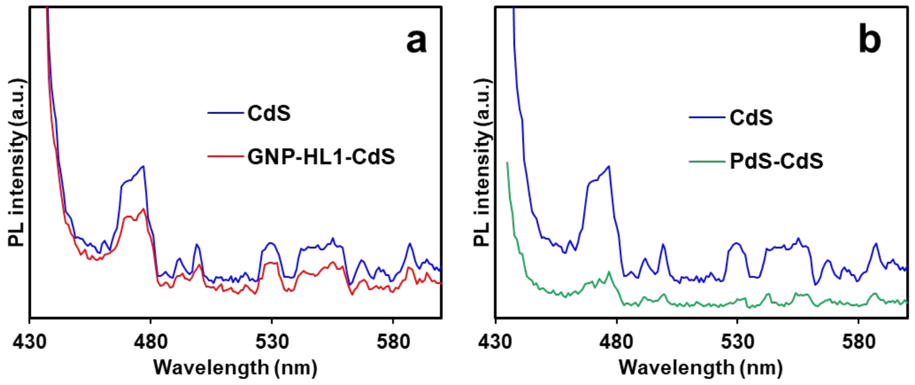

Figure S15. PL spectra ($\lambda_{excitation}$: 425 nm) of: a) CdS and GNP-HL1-CdS, b) CdS and PdS-CdS.

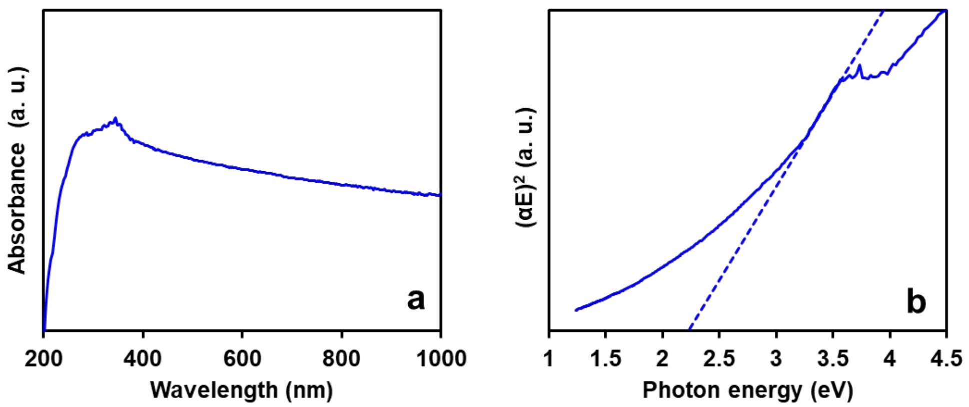

Figure S16. a) UV absorption spectrum of GNP-H_2L2-PdS-CdS; b) Plots of $(\alpha E)^2$ (direct transitions) vs the photon energy (E) for GNP-H_2L2-PdS-CdS.
